# The AMPK activator metformin improves recovery from demyelination by shifting oligodendrocyte bioenergetics and accelerating OPC differentiation

**DOI:** 10.3389/fncel.2023.1254303

**Published:** 2023-10-12

**Authors:** Mohanlall Narine, Maryam A. Azmi, Martin Umali, Ashley Volz, Holly Colognato

**Affiliations:** ^1^Department of Pharmacological Sciences, Stony Brook University, Stony Brook, NY, United States; ^2^Program in Neurosciences, Stony Brook University, Stony Brook, NY, United States

**Keywords:** metformin, oligodendrocyte, metabolism, cuprizone, multiple sclerosis, myelin repair

## Abstract

Multiple Sclerosis (MS) is a chronic disease characterized by immune-mediated destruction of myelinating oligodendroglia in the central nervous system. Loss of myelin leads to neurological dysfunction and, if myelin repair fails, neurodegeneration of the denuded axons. Virtually all treatments for MS act by suppressing immune function, but do not alter myelin repair outcomes or long-term disability. Excitingly, the diabetes drug metformin, a potent activator of the cellular “energy sensor” AMPK complex, has recently been reported to enhance recovery from demyelination. In aged mice, metformin can restore responsiveness of oligodendrocyte progenitor cells (OPCs) to pro-differentiation cues, enhancing their ability to differentiate and thus repair myelin. However, metformin’s influence on young oligodendroglia remains poorly understood. Here we investigated metformin’s effect on the temporal dynamics of differentiation and metabolism in young, healthy oligodendroglia and in oligodendroglia following myelin damage in young adult mice. Our findings reveal that metformin accelerates early stages of myelin repair following cuprizone-induced myelin damage. Metformin treatment of both isolated OPCs and oligodendrocytes altered cellular bioenergetics, but in distinct ways, *suppressing* oxidative phosphorylation and *enhancing* glycolysis in OPCs, but *enhancing* oxidative phosphorylation and glycolysis in both immature and mature oligodendrocytes. In addition, metformin accelerated the differentiation of OPCs to oligodendrocytes in an AMPK-dependent manner that was also dependent on metformin’s ability to modulate cell metabolism. In summary, metformin dramatically alters metabolism and accelerates oligodendroglial differentiation both in health and following myelin damage. This finding broadens our knowledge of metformin’s potential to promote myelin repair in MS and in other diseases with myelin loss or altered myelination dynamics.

## Introduction

Many disorders of the central nervous system (CNS) are accompanied by metabolic disturbances, with the resultant energy imbalances being linked to neuronal degeneration, cell death, and cognitive impairment. In the demyelinating disease Multiple Sclerosis (MS), immune cells attack myelinating oligodendrocytes (OL), destroying the myelin sheath that surrounds axons. Demyelination disrupts appropriate neuronal axon potential propagation, leading to functional disturbances, and failed myelin repair has devastating consequences for neuronal health that ultimately lead to neurodegeneration. A growing body of evidence indicates that MS, in part, is also a disease of significant metabolic dysfunction. In one example, the induction of hypoxia-induced factor 1α (HIF-1α), occurs early on in MS lesions, suggesting that hypoxia-induced metabolic dysfunction contributes to neuroinflammation in MS (Aboul-Enein et al., 2003; Lee et al., 2004; Halder and Milner, 2020). HIF-1α has been shown to a have a role in regulating cellular glycolysis when subjected to conditions of hypoxic stress (Zhang et al., 2018), and reduced oxygen delivery is known to occur in MS patients (Swank et al., 1983; Varga et al., 2009; Halder and Milner, 2020).

OLs in particular have unique metabolic requirements for proper functioning. A recent study demonstrated that under favorable and nutrient rich conditions, human-derived OPCs and OLs both rely significantly more on glycolysis than oxidative phosphorylation (OXPHOS) to generate ATP (Rone et al., 2016). When subjected to stress conditions like those observed in the demyelinating disease MS, OLs retract their processes to destabilize the compact axonal myelin sheath, which is thought to limit cell death by entering a less energy intensive, low metabolic state (glycolysis) (Ziabreva et al., 2010). This response is perhaps favorable in the short term, preserving existing OLs should they go on to experience conditions conducive for metabolic rebound. Therefore, it is possible that altering OL metabolism may prove to be beneficial to promote myelin repair, a highly energy intensive process.

Cells respond to changing energy demands using AMP-activated protein kinase (AMPK), the so-called metabolic sensor of the cell. This serine/threonine kinase senses changes in cellular metabolism to regulate activity or expression of proteins involved in ATP synthesis. During periods of metabolic stress (e.g., starvation, exercise, and ischemia), energy depletion leads to shifts in the ADP/ATP and AMP/ATP ratios, which are detected by AMPK and activate the enzyme complex to bias the cell into catabolic processes for ATP generation. In energy-replete conditions (where ATP levels are high), AMPK is kept in its inactive form to stimulate anabolic events such as lipogenesis, gluconeogenesis, and protein synthesis (Gowans et al., 2013).

Metformin, an anti-hyperglycemic drug used to treat Type II Diabetes Mellitus, is best known as an indirect activator of AMPK, although it can also mediate multiple non-AMPK-dependent effects (Foretz et al., 2014). Metformin can inhibit complex I function in the mitochondria, decreasing ATP output and therefore shifting the cellular AMP/ADP: ATP ratio detected by the AMPK γ-subunit (Zhou et al., 2001). Neumann et al. (2019) have recently shown that metformin is able to mitigate the poor differentiation capacity selectively found in aged OPCs, restoring their ability to respond to pro-differentiation cues. In contrast, while metformin treatment in younger mice has been reported to improve myelin outcomes following cuprizone- and lysolecithin-mediated myelin damage (Houshmand et al., 2019; Kosaraju et al., 2020; Sanadgol et al., 2020), the effect of metformin on cell metabolism and differentiation status in non-aged OPCs and OLs remains poorly understood. Here we set out to investigate whether extrinsic manipulation of OL metabolism by metformin could directly influence the ability of non-aged OPCs to differentiate and subsequently myelinate, as well as investigate metformin’s ability to modulate OPC/OL bioenergetics across the differentiation process. Our findings reveal that metformin treatment in both OPCs and OLs alters cellular bioenergetics to promote differentiation in both healthy OPCs and OLs in an AMPK-dependent manner, and accelerates the repair process following cuprizone-induced myelin damage in young adult mice when administered at endogenous remyelination onset.

## Materials and methods

### Animals

All animal procedures were in compliance with the National Institute of Health Guide for the Care and Use of Laboratory Animals and approved by the institutional animal use and care committee (IACUC) at Stony Brook University. Female C57/BL6 mice (Envigo) were housed in groups of 3 or 4 per cage under standard 12-h light and dark cycle with uninterrupted access to food and water. Mice were grown to 8 weeks of age before switching to a 0.2% cuprizone diet (Envigo) in their feed. After 5 weeks, cuprizone infused chow was withdrawn and replaced with normal mice feed for 1.5 weeks. In metformin treated mice, U.S. pharmaceutical grade metformin hydrochloride (Sigma-Aldrich, Cat. No. 1396309) was added to drinking water at a dose of 250 mg/kg or 500 mg/kg with daily water intake in all mice being ~3 mL per day beginning at week 4 of the cuprizone treatment period (mice were therefore treated with metformin for a total of 2.5 weeks). Animals were anesthetized with isoflurane prior to transcardial perfusion with 4% paraformaldehyde (PFA; Sigma-Aldrich) and brain dissection.

### Primary cell cultures

Timed-pregnant female Sprague–Dawley rats were obtained from Envigo. Cortices were removed from pups between postnatal day 0–2, followed by removal of the meninges and cortical digestion using papain (Worthington). Digested cortices were then placed in 75cm^2^ tissue culture flasks containing Dulbecco’s modified eagle media (DMEM-Corning Cellgro) supplemented with 10% fetal bovine serum and 1% penicillin and streptomycin (Gibco). These mixed glia cultures were grown for 14 days at 37°C and 7.5% CO_2_ with media changes every 3–4 days. On the 14^th^ day, OPCs were isolated by mechanical dissociation followed by differential adhesion where the cell suspension was plated on uncoated petri dishes to allow for microglia separation. Following differential adhesion, isolated OPCs were first plated in tissue culture treated dishes (Thermo Fisher) coated with poly-d-lysine (Sigma-Aldrich) in SATO media +10 ng/mL PDGFRα and 10 ng/mL FGF (Peprotech) to establish a similar baseline for all OPCs in culture. At 24-h post-plating, OL differentiation was induced using SATO media +400 ng/mL triiodothyronine (T3) +0.5% FBS. OL cultures were allowed to differentiate for up to 7 days at 37°C with 7.5% CO_2_. Daily half media changes were performed on OL cultures to obtain either 0, 200, 500 or 1,000 μM final concentration metformin.

### Protein lysis and western blot

OPCs and OLs cells were lysed in a 20 mM Tris and 1% sodium dodecyl sulfate (SDS) pH 7.4 solution pre-heated to 95°C. Using a cell scraper, lysate was collected into microfuge tubes and placed on a 95°C heating block for 10 min. Protein concentration was determined following the manufacturer’s instructions of the Bio-Rad DC Assay (Biorad). Protein lysates were electrophoresed on 10 or 12% SDS polyacrylamide gels (Biorad). After SDS-PAGE, proteins were transferred to a 0.45 μm nitrocellulose membrane (GE Healthcare) using western blotting, and blocked with a 4% bovine serum albumin (Sigma-Aldrich) in 1x tris buffered saline +0.1% tween (Sigma-Aldrich) solution (TBST). After blocking, the membrane was incubated overnight with the following primary antibodies: 1:750 rabbit AMPKα (Cell Signaling, 5,831), 1:750 rabbit phospho-AMPKα (Thr172) (Cell Signaling, 2,531), 1:750 rabbit ACC (Cell Signaling, 3,676), 1:750 rabbit phospho-ACC (Ser79) (Cell Signaling, 11,818), and 1:750 rabbit Histone H3 (Santa Cruz Biotenchnology, FL-136). The next day the primary antibodies were washed 3 times with 1x TBST. Next, membranes were incubated in 1x TBST for 1 h with 1:2000 anti-rabbit horseradish peroxidase linked secondary antibodies (Cell Signaling, 7,074). Enhanced chemiluminescence substrate kit (Millipore) was used to develop protein blots on radiography films (Thermo Scientific).

### Oxygen consumption rate and extracellular acidification rate measurement

Oxygen consumption rate (OCR) and extracellular acidification rate (ECAR) measurements were taken on the Seahorse XFe^96^ platform from Agilent. Seahorse 96-well plates (Agilent) were first coated with Cell-Tak (Corning) at 4 μg/cm^2^, followed by the addition of primary OPCs in SATO media + T3 to induce differentiation at 37°C and 7.5% CO_2_. As in above differentiation assays, a daily half media change was performed without metformin or with metformin at 200, 500, 1,000 μM. For OCR measurements, the cell mitochondria stress test assay (Agilent) was performed using 2 μM oligomycin, 2 μM FCCP, and 0.5 μM rotenone/antimycin A loaded into each delivery port. 1 h prior to running the assay, all media was removed and replaced with the Seahorse DMEM media supplemented with 2 mM L-glutamine (Sigma-Aldrich), 25 mM dextrose (Fisher Scientific), and 1 mM pyruvate (Fisher Scientific). Cells were kept in a 37°C with no CO_2_ for 1 h prior to assay run. OCR measurements were normalized to total DNA content using the CyQuant proliferation assay (Invitrogen) according to the manufacturer’s instructions. For ECAR measurement, the glycolysis stress test kit (Agilent) was performed, utilizing 10 mM glucose, 2 μM oligomycin, and 50 mM 2-DG. ECAR measurements were also normalized to total DNA content.

### Immunocytochemistry and immunohistochemistry

Immunocytochemistry (ICC, for *in vitro*) and immunohistochemistry (IHC, for *in vivo*) were performed to detect the presence of oligodendroglia, microglia, or astrocyte markers. The OPC markers NG2 [Millipore; mouse (MAB5384) or rabbit (AB5320)] and PDGFRα [R&D Systems; goat (AF1062)], pre-myelinating and myelinating OL marker CNP [Sigma Aldrich; mouse (C5922) [1:50] or Novus Biologicals, chicken (NB100-1935, for *in vitro* ICC experiments), myelin marker MBP [Biorad; rat (MCA409S)], OL marker ASPA [Millipore; rabbit (ABN1698)], OL lineage marker Olig2 transcription factor [Millipore; mouse (MABN50), rabbit (AB9610)], microglia marker Iba1 [Wako; rabbit (019–19,741)], and astrocyte marker GFAP [Abcam; chicken (AB4674)] were diluted in 5% donkey serum (Sigma-Aldrich) with 0.5% Triton-X (Sigma-Aldrich), 1x phosphate buffered saline (PBS) and 0.02% sodium azide. The proliferation marker ki67 (Invitrogen 14-5698-82), was also used under the same conditions. Brain tissue was embedded in optimal cutting temperature compound (Sakura Finetek United States) and sectioned at 30 μm. Sections were placed in cryoprotectant solution and kept at −20°C until time of use. Immunostaining was carried out by incubating primary antibodies (used at dilution of 1:100 unless stated otherwise) in blocking buffer overnight at 4°C on a shaker. The following day unbound primary antibodies were washed off with 1x PBS at room temperature. Incubation with species appropriate secondary antibodies conjugated to Cy5, Cy3, Alexa 488 (Jackson Laboratories) or Alexa 680 (Thermo Scientific) was carried out at a concentration of 1:250 in above blocking buffer for 1 h at room temperature. 1x PBS was used to wash and remove unbound secondary antibodies. DAPI stain was used to visualize cell nuclei. Immunostained coverslips with cells or tissue sections were then mounted on microscope slides with SlowFade gold (Thermo Scientific) media to preserve fluorescent signal.

### TUNEL assay

TUNEL (Roche) was performed according to manufacturer’s protocol with a slight modification to allow for co-staining with the oligodendroglial markers PDGFRα and ASPA. Tissue sections were first blocked in 5% donkey serum with 0.2% Triton-X and 1x PBS with 0.02% sodium azide. Primary antibodies (PDGFRα and ASPA) were used at 1:100 and incubated overnight at 4°C. After washing unbound primary antibodies with 1x PBS, tissue sections were incubated with 4% PFA at room temperature for 20 min. After removal of PFA, sections were further permeabilized with 0.1% Triton-X (v/v) + 0.1% sodium citrate (w/v) solution for 2 min on ice. Afterwards, the TUNEL solution was added according to the manufacturer’s protocol. Lastly, sections were incubated with Alexa 488 and Cy3 (both at 1:250) antibodies, and DAPI prior to mounting in SlowFade gold medium.

### Microscopy

All fluorescent imaging was performed using the Leica TCS SP8X confocal microscope system and the Leica Application Suite (LAS X) software. Images were taken at either 20x or 40x magnification using oil immersion objectives. A total of 9 fields per section were taken with an optical resolution set at either 1 μm (for 40x) or 2 μm (with 20x) in the z–axis. Image acquisition parameters were unchanged within individual experiments to determine differences in signal intensities.

### AMPK siRNA

Nucleofection of isolated OPCs was performed with siRNA mixtures to target both AMPKα_1_ (Dharmacon, Cat. No. L-091373-02-0005) and AMPKα_2_ (Dharmacon, Cat. No. L-100623-02-0005), using the basic nucleofector kit for primary mammalian glial cells (Lonza Biosciences). Control cells were transfected with a pool of non-targeting siRNAs (Dharmacon). OPCs were grown in high growth factor media (1x SATO with 20 ng/mL PDGFRα and 20 ng/mL FGF) at 37°C + 7.5% CO_2_ for 24 h prior to transfection. After trypsinization, cells were spun down and resuspended in the nucleofector resuspension solution according to the manufacturer’s protocol. Cells were then returned to the 37°C + 7.5% CO_2_ incubator for 1 to 1.5 h prior to siRNA transfection with 8 μL of AMPKα_1_ siRNA at a stock concentration of 20 μM and 6 μL of AMPKα_2_ siRNA at a stock concentration of 20 μM. Post-transfection, cells were immediately resuspended in the high growth factor media and plated in tissue culture treated petri dishes for 24 h after which the media was changed to OL differentiation media (1x SATO + T3).

### Metabolic inhibition

OXPHOS was inhibited by treating oligodendrocytes *in vitro* with 1 μM of oligomycin (Agilent) to inhibit complex I and induce deficits in mitochondrial activity. 5 mM 2-DG was used to inhibit glycolysis.

### Analysis

Intensity analysis was conducted with ImageJ (NIH). Images were thresholded at a level capable of removing background signal. The same threshold algorithm was applied to all images within an experiment. Regions of interest (ROI) for analysis were manually selected by drawing boundaries based on callosum borders, readily apparent in DAPI-stained images as a marked change in cell density. Mean values within ROI and area percentages from total ROI were calculated. Cell counts were done in LAS X on optical stacks. Statistics were performed using GraphPad Prism 9.0. Where applicable, unpaired Student *t*-tests, Welch’s *t*-test, one-way and two-way ANOVA were performed on parametric data sets, and Mann–Whitney test was performed on non-parametric data. Statistical significance was defined as a value of *p* < 0.05. All analysis was performed on data from biological replicates.

## Results

### AMPK is highly active during oligodendrocyte differentiation

Before attempting modifications, we first sought to determine AMPK levels and its activity as newly-formed oligodendrocytes (OLs) differentiated. OPCs generated from rodent primary cell cultures were grown in differentiation media for 7 days in which they progress from a mixture of OPCs and newly-formed OLs (at day 1) to mature, myelin producing OLs (at day 7). Protein lysates were collected at each time point and levels of phosphorylated AMPKα (which represents active AMPK) and total AMPKα were assessed (Supplementary Figure S1). AMPKα protein levels were high at early stages of differentiation (day 1) but also significantly (*p* < 0.05) increased as differentiation proceeded to day 5 (Supplementary Figure S1C). However, the ratio of phosphorylated AMPKα (pAMPKα) relative to total AMPKα significantly decreased during oligodendrocyte differentiation (Supplementary Figure S1G), indicating changes in the active AMPK. We next investigated the phosphorylation of ACC, a downstream target of activated AMPK. While total ACC levels did not significantly change, the overall level of pACC relatve to control protein Histone H3 increased by day 7 differentiation (Supplementary Figure S1D; *p < 0.05), the levels of phosphorylated ACC (pACC) and the ratio of pACC/ACC did not significantly change during OL differentiation (Supplementary Figures S1D,F).

### Glycolysis increases as oligodendrocytes differentiate

**Figure 1:**
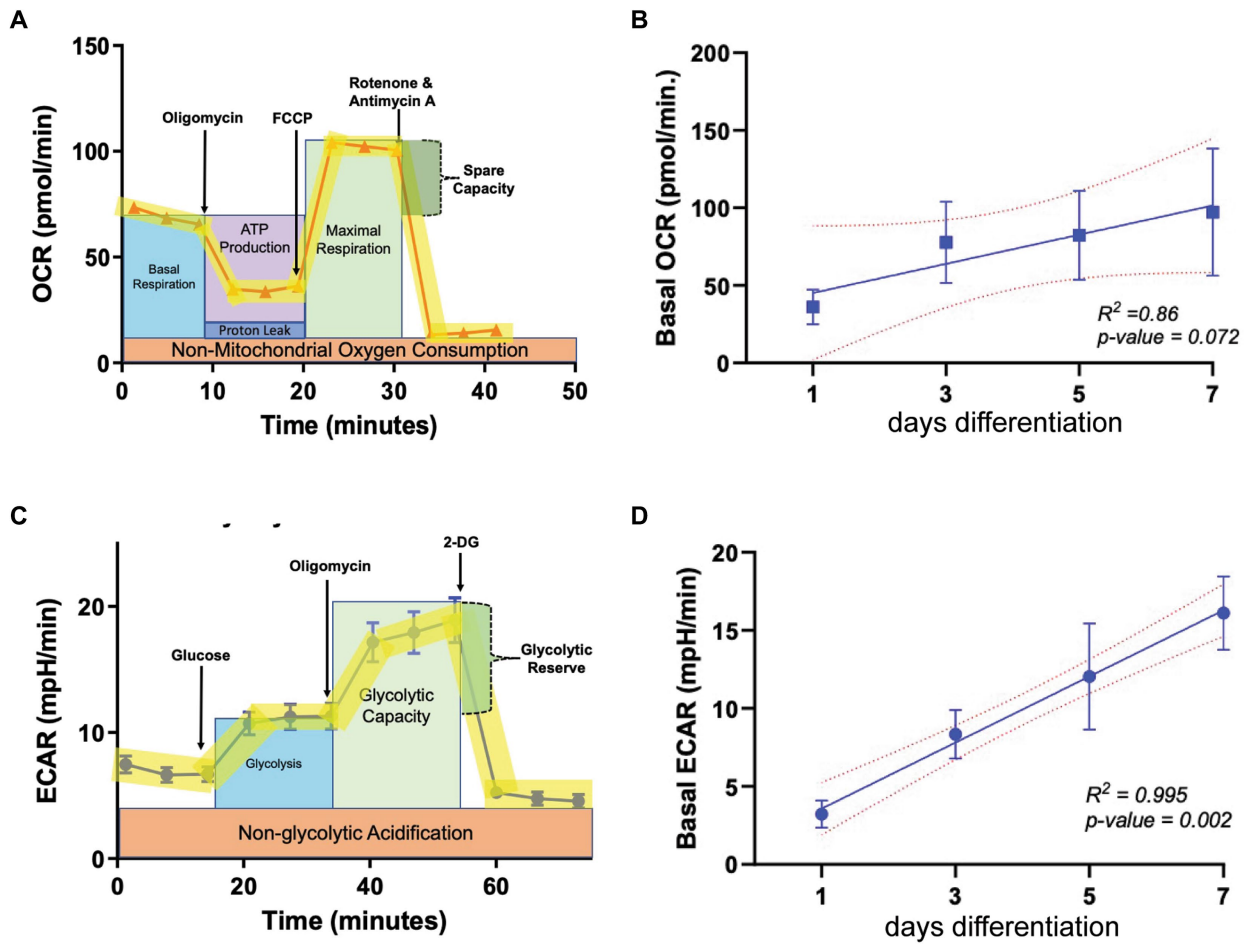
Metabolic parameters in oligodendroglia during differentiation. **(A)** Example profile of the mitochondria stress test assay used to determine oxygen consumption rate (OCR, representing mitochondrial respiration) at multiple time points during OL differentiation (day 1, 3, 5, and 7). **(B)** Baseline OCR during OL differentiation (*n* = 3). **(C)** Example profile of the glycolysis stress test used to determine extracellular acidification rate (ECAR, representing glycolytic output) at multiple time points during OL differentiation (day 1, 3, 5, and 7). **(D)** ECAR during OL differentiation (*n* = 3). Error bars represent standard error of the mean.

To determine baseline OL bioenergetics during the cellular differentiation process, the oxygen consumption rate (OCR) and extracellular acidification rate (ECAR) were calculated using the Seahorse live cell-based assay, in which the application of metabolic stressors helps to determine the level of mitochondrial respiration and glycolysis, respectively (Figures 1A,C). OPCs were allowed to differentiate to mature oligodendrocytes (OLs) across a 7-day period in differentiation-promoting medium (SATO+T3), and OCR and ECAR were determined every 2 days beginning at day 1. Our regression analysis revealed that OCR levels increase as OPCs differentiate in a linear manner, evident by a *R*^2^ value of 0.86 (Figure 1B) although the correlation is not statistically significant due to variability among biological replicates. ECAR also increased in a linear fashion as OLs differentiated (Figure 1D) but in contrast to OCR, there was a significant 4-fold increase (*p* = 0.002) in levels over the 7-day differentiation period. This robust increase in ECAR during OL differentiation is consistent with previous reports suggesting that mature, myelinating OLs increasingly rely on glycolysis to maintain cellular functions (Fünfschilling et al., 2012; Rao et al., 2017).

### Metformin increases oligodendrocyte AMPK activation in a dose-dependent manner

**Figure 2:**
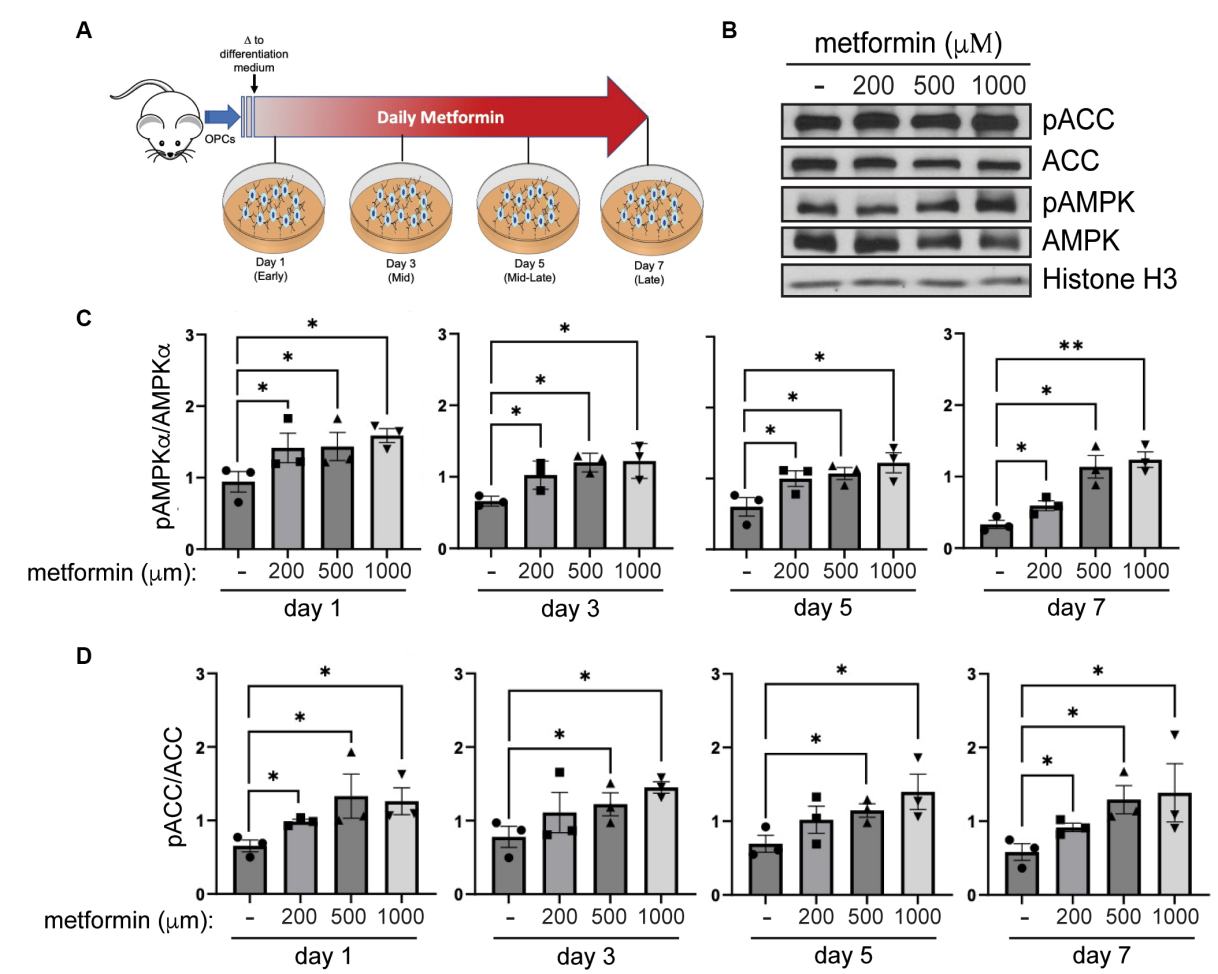
AMPK activity increases in metformin-treated oligodendrocytes. **(A)** Metformin treatment schematic for OPCs during a 7 day differentiation time course. **(B)** Representative western blot of pACC, ACC, pAMPKα, AMPKα, and Histone H3 (loading control) in OLs treated with 200 μM, 500 μM, and 1 mM metformin at day 7 differentiation. **(C)** Quantification of pAMPKα/AMPKα in metformin-treated OLs at 1, 3, 5, or 7 days of differentiation. **(D)** Quantification of pACC/ACC (downstream target of activated AMPK) in metformin-treated OLs at 1, 3, 5, or 7 days of differentiation. Statistical analysis was performed using one-tailed Mann–Whitney test, or unpaired *t*-test with or without Welch’s correction. *n* = 3. *p ≤ =0.05, **p ≤ 0.01. ns = not significant.

Depending on the regulatory agencies overseeing its clinical use, the maximum oral dose of metformin administered per person per day ranges between 2,250–3,000 mg with a regimen of 500 mg to 1,500 mg twice daily (taken every 12 h; Bailey and Turner, 1996; Kanto et al., 2017). Using these values, along with data from studies measuring metformin concentrations in rodents, a physiologically based metformin pharmacokinetic model was established for estimating metformin concentrations in various human tissues (Zake et al., 2021). This model revealed that the maximum metformin concentration (C_max_) achieved *in vivo* is within the kidneys (the site of excretion) at ~2,200 μM. In the intestines, where metformin is absorbed, C_max_ is ~1,250 μM and in the liver, the main site for metformin distribution, C_max_ was found to be ~200 μM with the half-life ranging between 3 and 5 h (Zake et al., 2021). On this basis, we prepared primary OPC cultures from rodent pups and treated them *in vitro* daily with either 200 μM, 500 μM, or 1 mM metformin and compared them to vehicle (no metformin)- treated cells (Figure 2A).

To assess AMPK activation in response to metformin dosing, we performed western blot analysis to determine levels of pAMPKα and AMPKα, and phosphorylation of AMPK’s downstream target, ACC (Figure 2B). Activation of AMPKα (indicated by increased AMPKα phosphorylation) leads to phosphorylation of ACC, which in turn inhibits ACC action. Hence, if metformin is capable of activating AMPK in OLs, we would expect to see inhibition of ACC reflected in increased ACC phosphorylation (pACC). Our protein analysis revealed that metformin treatment significantly increased pAMPKα phosphorylation (pAMPKα/AMPKα) in a dose dependent manner (Figure 2C). In conjunction, we observed a significant increase (Figure 2D) in ACC phosphorylation (pACC/ACC). This enhanced AMPK activation/ACC inhibition pattern was consistent throughout all time periods of metformin treatment (day 1 to day 7), implying that metformin is capable of acting on oligodendroglia throughout lineage progression.

### Metformin treatment alters oligodendroglial energetics at all stages of differentiation

**Figure 3:**
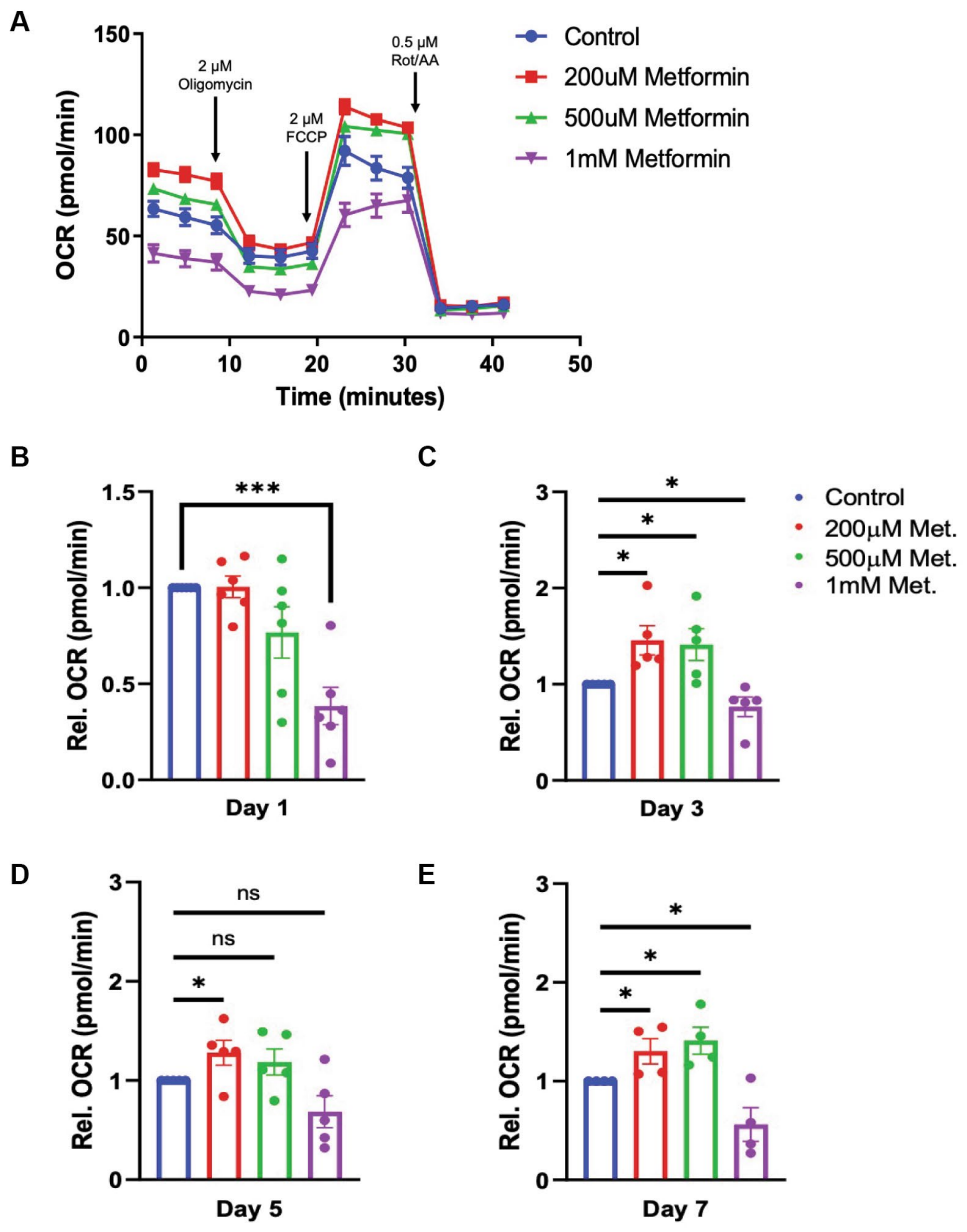
Oxygen consumption rate (OCR) in metformin-treated oligodendrocytes. **(A)** Representative OCR over time plot of control and metformin-treated OLs undergoing the mitochondrial stress test using oligomycin, FCCP, and rotenone/antimycin A. **(B–E)** Quantification of relative OCR in OLs at differentiation day 1, 3, 5, and 7. Statistical analysis was performed using one-tailed Welch *t*-test. n = 5. *p ≤ =0.05, ***p ≤ 0.001. ns = not significant.

Given that metformin treatment increased AMPK activity (Figure 2), we predicted that metformin would alter OL metabolism. As in experiments on untreated OLs (Figure 1) the Seahorse live cell-based assay was used to transiently subject OLs undergoing daily metformin treatment to the mitochondrial stressors oligomycin, FCCP, rotenone, and antimycin A (Figure 3A). These drugs target varying components of the electron transport chain (ETC) and by measuring the OCR response, we obtain data related to the level of OXPHOS and rate of ATP production occurring in the mitochondria. At day 1 of differentiation, the high dose of metformin (1 mM) selectively caused OLs to experience a *decrease* in OCR (***p < 0.001; Figure 3B). However, by day 3 and continuing on through day 7, OCR levels increased in response to 200 μM and 500 μM metformin (*p < 0.05; Figures 3C–E). In contrast, throughout most of the OL differentiation process (at day 1, day 3, and day 7), a significant decrease in OCR was observed with 1 mM metformin treatment (***p < 0.001, *p < 0.05).

**Figure 4:**
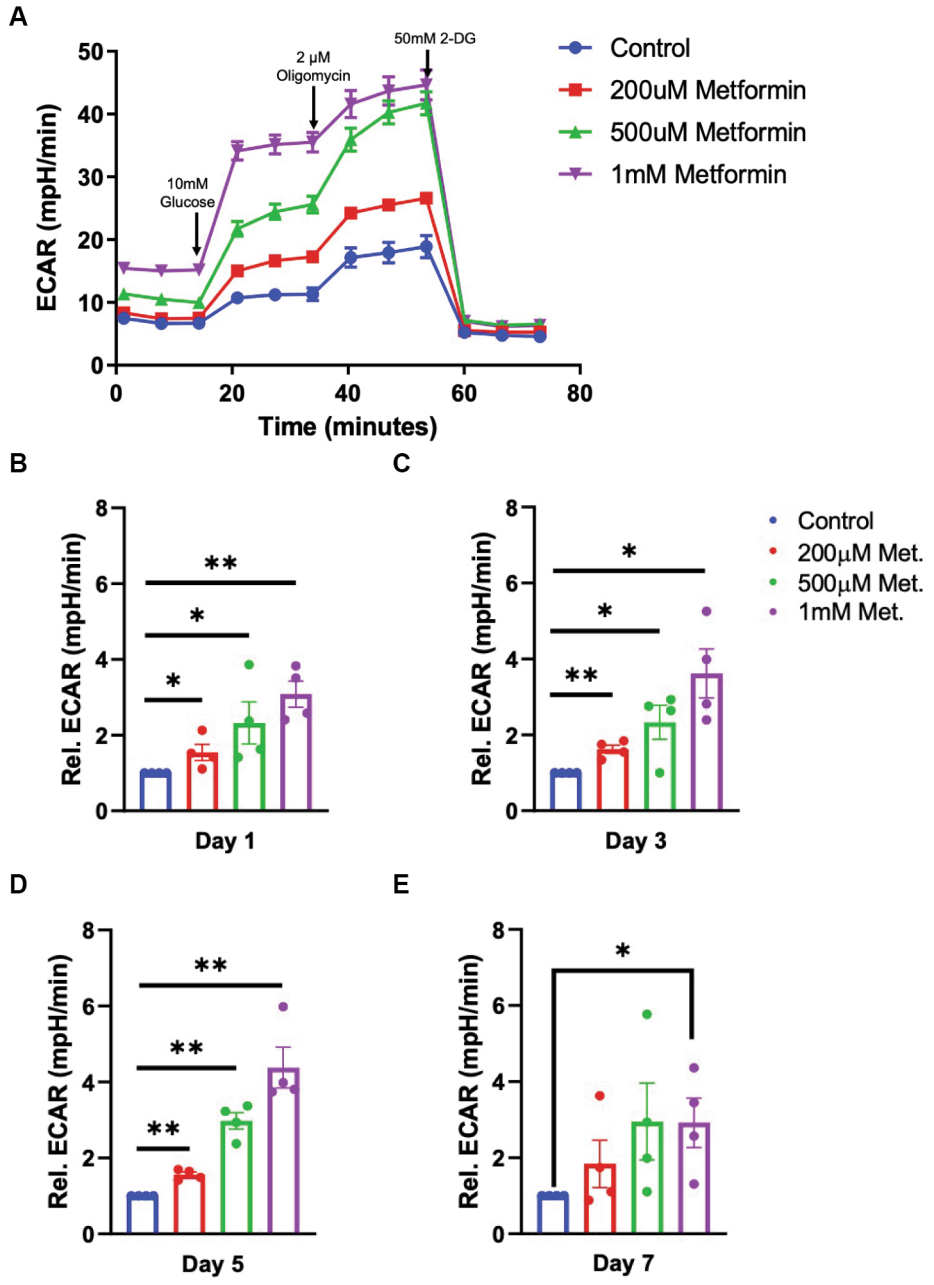
Extracellular cellular acidification rate (ECAR) in metformin-treated oligodendrocytes. **(A)** Representative ECAR over time plot of control and metformin-treated OLs undergoing the glycolysis stress test with glucose, oligomycin, and 2-DG. **(B–E)** Quantification of relative ECAR in OLs at differentiation day 1, 3, 5, and 7. Statistical analysis was performed using one-tailed Welch *t*-test. *n* = 5, *p ≤ =0.05, **p ≤ 0.01. ns = not significant.

Next, using a glycolysis stress test, we introduce glucose, oligomycin and 2-deoxy-d-glucose (2-DG) at specific times during the assay to target the OL glycolytic pathway (Figure 4). By measuring the changes in the ECAR, we are able to determine how metformin treatment alters OL glycolysis (the second ATP producing pathway). In contrast to OCR readings, the glycolysis stress test revealed that ECAR levels significantly increased with metformin administration in a dose dependent manner at days 1, 3, and 5 of differentiation (*p < 0.05, **p < 0.01; Figures 4A–D). However, while ECAR trended toward higher levels at day 7, a statistically significant increase in ECAR was only observed upon the highest (1 mM) metformin dose (*p < 0.05; Figures 4A,E).

### Metformin accelerates oligodendrocyte differentiation

AMPK activation by metformin enhances *both* OXPHOS and glycolysis in OLs, as indicated by the increases in OCR and ECAR, respectively (Figures 3, 4). This “bioenergetic phenotype” (denoted by increases in *both* OXPHOS and glycolysis) has been shown to be necessary for varying cellular functions such as the differentiation of osteoclasts and for supporting the biosynthetic pathways involved in the macrophage accumulation and interleukin-1β production, within white adipose tissue (Li et al., 2020; Sharma et al., 2020). Therefore, we examined whether the enhanced bioenergetics observed in metformin-treated OLs would lead to changes in OL phenotypes. Given the large amounts of energy required for OL development and myelination, we hypothesized that the increases in both OXPHOS and glycolysis from metformin treatment might alter OL differentiation dynamics.

**Figure 5:**
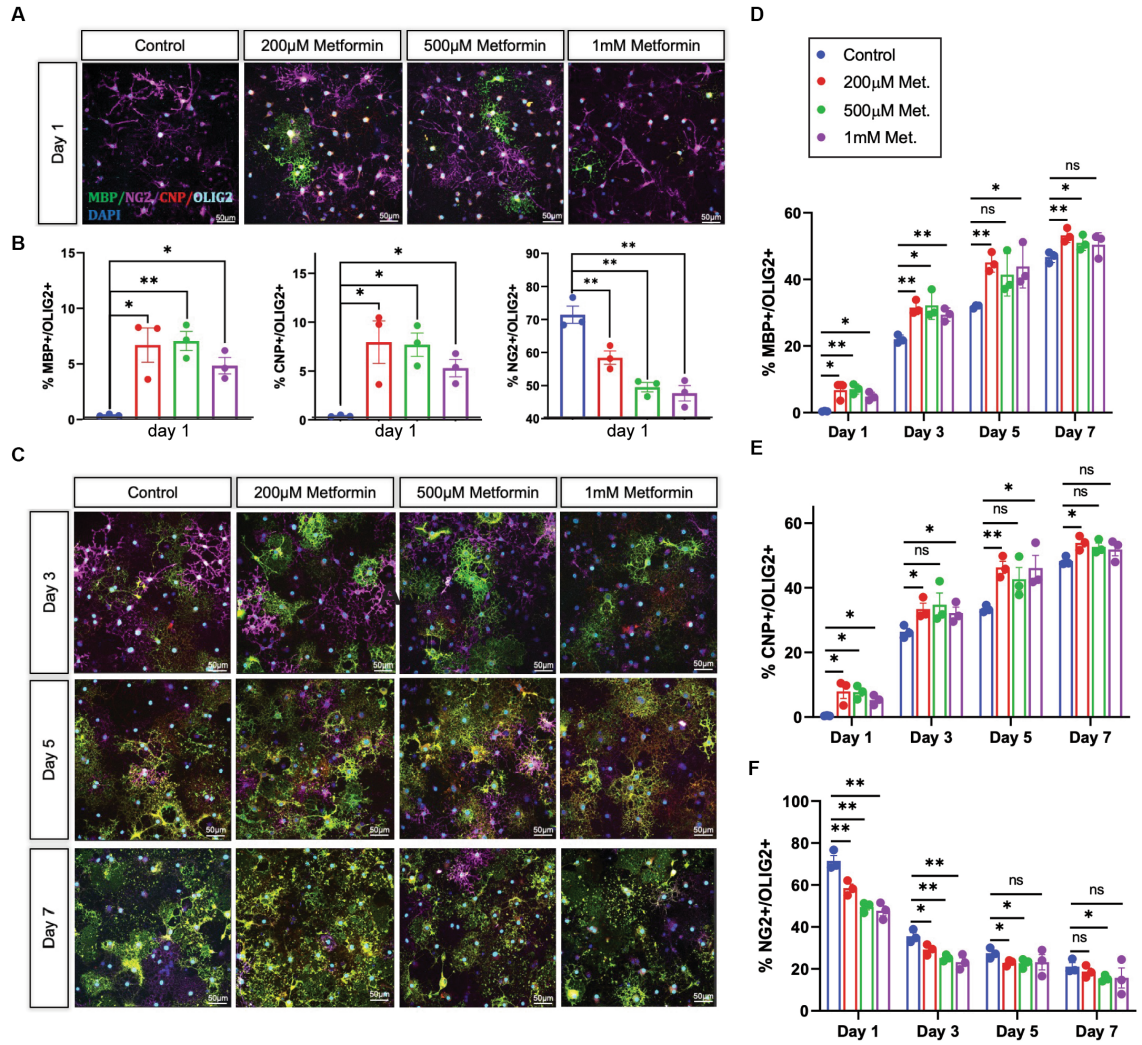
Oligodendrocyte lineage stage markers are altered in response to metformin. **(A)** OPCs subjected to differentiation medium for 1 day +/− metformin were fixed and stained with the lineage stage specific antibodies to detect NG2 (magenta; OPCs), CNP (red; both immature and mature OLs), and MBP green; mature OLs. Shown are representative confocal micrographs at differentiation day 1. **(B)** Metformin-treated OLs showed substantial changes in the percentages of MBP+, CNP+, and NG2+ cells at differentiation day 1. **(C)** Representative confocal micrographs at differentiation day 3, 5, and 7. Mean percentages of Olig2+ cells that express MBP **(D)**; CNP **(E)**, and NG2 **(F)** at all differentiation days. Statistical analysis was performed using one-way ANOVA with multiple comparisons (DF = 8 within columns). *n* = 3. *p ≤ =0.05, **p ≤ 0.01. ns = not significant.

OLs were treated with either 0 (controls), 200 μM, 500 μM, or 1 mM metformin daily, using half media changes with the appropriate concentration of metformin. OLs were fixed in 4% paraformaldehyde at day 1, 3, 5, and 7, the same timeline used in our metabolic analysis. After fixation, immunocytochemistry was performed to detect lineage stage-specific proteins such as NG2 (early), CNP (mid and late), and MBP (late) to determine the degree of OL differentiation (Figure 5A). When compared to controls, we observed increases in the percentages of CNP+ and MBP+ cells (out of total Olig2+ cells) in OL cultures at day 1 in response to metformin treatment (Figure 5B). Concurrently, this increase in the percentage of MBP+ cells was accompanied by decreases in the percentages of NG2+ cells (i.e., OPCs) out of total Olig2+ cells (Figure 5B). Similarly, this pattern of increased CNP/MBP and decreased NG2 was observable for all other time points with metformin treatment (Figures 5C–F; Supplementary Figure S2 for single channel views). Interestingly, OLs given metformin at 200 μM and 500 μM resulted in the greatest increases in CNP+ and MBP+ cells (Figures 5D,E) whereas, the 1 mM metformin treated OLs saw the largest decrease in NG2+ cells (Figure 5F), with only a modest elevation in CNP+ and MBP+ cells (Figures 5D,E). However, the number of cells expressing the pan-oligodendroglia marker, OLIG2, remained unchanged throughout (Supplementary Figure S3) suggesting that metformin treatment, at all doses, accelerates OL differentiation (and does not change overall cell number during differentiation).

### Metformin requires AMPK to accelerate oligodendrocyte differentiation

**Figure 6:**
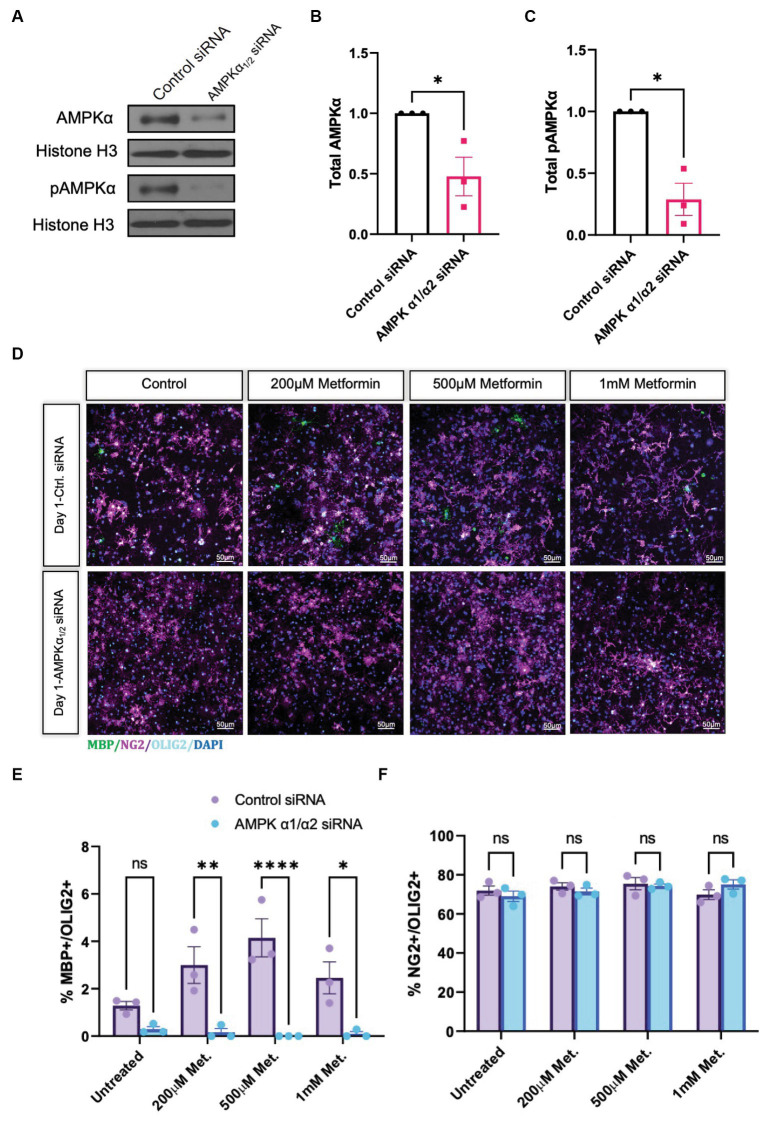
Metformin requires AMPKα_1/2_ to accelerate OL differentiation. **(A)** Representative western blot depicting AMPKα and pAMPKα in OLs transfected with either nontargeting siRNAs (control) or combined AMPKα_1/2_ siRNAs. **(B)** Quantification of relative AMPKα levels in either control or AMPKα_1/2_ siRNA conditions. **(C)** Quantification of relative pAMPKα levels in either control or AMPKα_1/2_ siRNA conditions. **(D)** Representative confocal micrographs of control siRNA or AMPKα_1/2_ siRNA transfected OLs following treatment with increasing doses of metformin for 1 day. **(E)** Percentage MBP-positive OLs at day following treatment with increasing doses of metformin, in either control siRNA or AMPKα_1/2_ siRNA transfected OLs. **(F)** Percentage of NG2-positive cells at day 1 following treatment with increasing doses of metformin, in either control siRNA or AMPKα_1/2_ siRNA transfected OLs. Statistical analysis was performed using one-tailed Welch T-test. *p < 0.05; **p < 0.001; ***p < 0.001. *n* = 3. Error bars represent standard deviations.

Metformin treatment leads to an increased percentage of cells that express the mature OL markers CNP and MBP, and thus is sufficient to promote OL differentiation *in vitro*. However, because of the pleiotropic effects of metformin and its possibility to act independently of its effects on AMPK (Israelsen and Vander Heiden, 2015; Cuyàs et al., 2018; Atawia et al., 2019), we next sought to determine if metformin’s ability to promote OL differentiation was AMPK-dependent. We performed siRNA knockdown to prevent AMPK expression, then analyzed the effect of metformin on OL cultures after 1 day of differentiation, the time point where the most robust effect of metformin on OL differentiation was observed (Figure 5B). Using either a pool of nontargeting siRNAs for a control transfection, or a mixture of two siRNA pools to target the two isoforms of the catalytic AMPK α subunit α1 and α2, both of which are expressed in OLs (Zhang et al., 2014), we reduced AMPKα levels in OLs (Figures 6A,B), leading to a significant reduction in both AMPK and pAMPKα in the cell (Figures 6B,C). We additionally used immunocytochemistry to detect NG2 (OPCs), and MBP (mature OLs; Figure 6D). Cell counts revealed that, compared to the control siRNA group, metformin treatment was unable to promote the expression of MBP following knockdown of AMPKa (Figure 6E), indicating that AMPK activity is necessary for metformin to accelerate OL differentiation. Furthermore, the number of OPCs were unchanged in both control and AMPKα_1/2_ siRNA groups (Figure 6F).

### Metformin’s ability to accelerate oligodendrocyte differentiation depends on normal metabolic function

**Figure 7:**
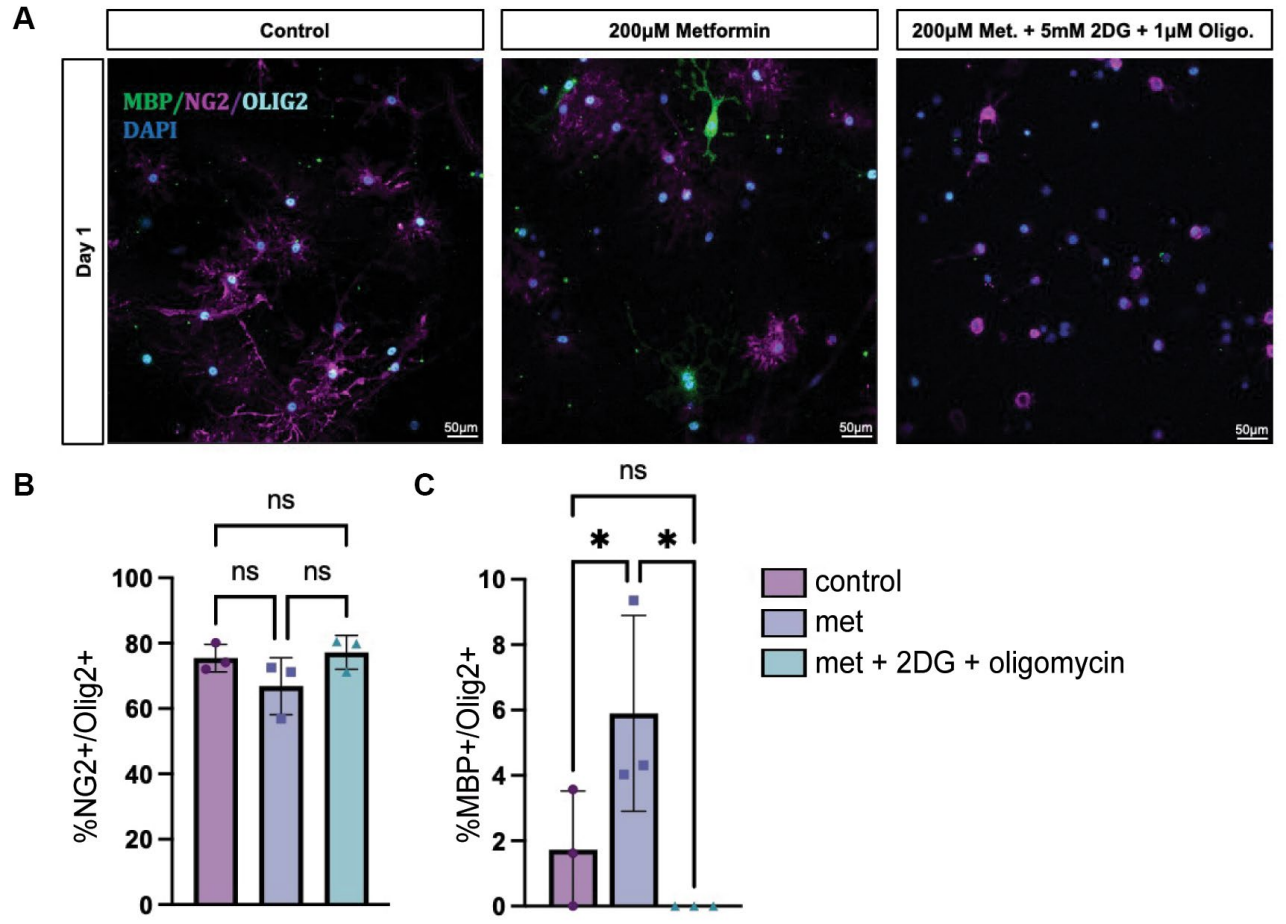
Inhibiting OXPHOS and glycolysis prevents metformin’s ability to induce oligodendrocyte differentiation. **(A)** Representative images from lineage stage-specific immunocytochemistry in day 1 OL cultures (MBP (green), NG2 (magenta), Olig2 (cyan), DAPI (blue)). **(B)** Quantification of Olig2+ cells that co-express NG2 or **(C)** MBP. 5 mM 2-DG and 1 μM oligomycin treatment was used to downregulate glycolysis and OXPHOS, respectively. Statistical analysis was performed using one-way ANOVA with multiple comparisons (DF = 6 within columns). *n* = 3 per condition. *ns*, not significant; *p < 0.05 Error bars represent standard error of the mean.

While we determined that metformin had the ability both to accelerate OL differentiation in an AMPK-dependent fashion (Figure 6) and to promote changes in OL bioenergetics (Figures 3, 4), it remained unclear whether metformin’s effects on differentiation were dependent on metformin’s ability to influence cellular metabolism. Therefore, we designed an experiment to block both OXPHOS and glycolysis in metformin-treated OLs by using 1 μM oligomycin and 5 mM 2-DG, respectively, and evaluated the cells after 1 day in differentiation-promoting medium (the time point at which metformin had the largest effect on differentiation). As expected, all groups had mostly NG2+ cells after 1 day in differentiation-promoting medium (Figures 7A,B). And, as before, the 200 μM metformin treated cells exhibited a significant increase in MBP+ cells at day 1 (Figure 7C). However, when metformin-treated cells were additionally treated with the metabolic inhibitors 2-DG and oligomycin, metformin treatment did not induce differentiation (Figure 7C).

### Metformin accelerates early myelin repair following cuprizone-mediated demyelination

Metformin is able to cross the BBB and has effects on the CNS, although the exact mechanism and sites of action are not fully known (Moreira, 2014). Considering metformin’s ability to activate AMPK, enhance OL bioenergetics, and induce OL differentiation *in vitro*, we sought to determine whether the same could occur *in vivo* during myelin repair. Several mouse models are readily available to study cellular repair responses in MS. In experimental autoimmune encephalomyelitis (EAE) an external immunization step is required to induce sensitization of myelin antigens, such as myelin oligodendrocyte glycoprotein (MOG), using an adjuvant that usually comprises of bacterial components to activate the immune system. In this way, T reactive myelin cells and anti-myelin antibodies are generated to induce demyelination (Constantinescu et al., 2011). However, to better understand the effect of metformin on myelin repair in the absence of metformin’s known effect on T cells (Nath et al., 2009; Paintlia et al., 2013a,b; Sun et al., 2016), we used cuprizone to induce demyelination. When administered at a specific concentration of 0.2% into animal feed for 5 weeks, cuprizone induces OL cell death, causing demyelination to occur, most prominently in the white matter tracts of the corpus callosum (Schmidt et al., 2013). Return to normal chow allows endogenous remyelination to occur, with myelin levels reaching that of control 5 weeks after cuprizone withdrawal, thereby mimicking the de/remyelination that is seen in relapse-remitting MS (Gudi et al., 2014).

**Figure 8:**
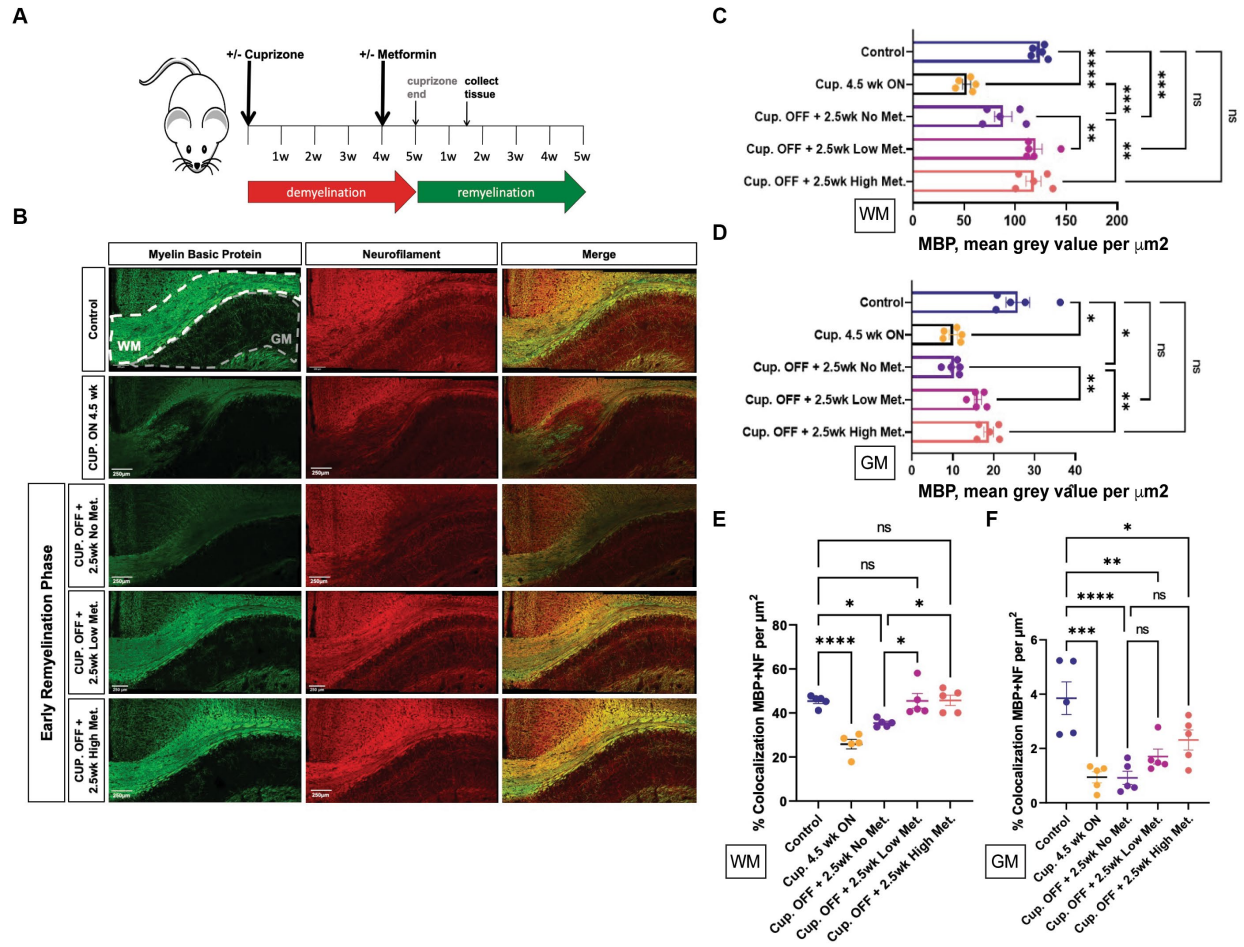
Metformin enhances early myelin repair following cuprizone-mediated demyelination. **(A)** Experimental paradigm for inducing de/remyelination in mice. Using the copper chelating agent cuprizone, 8 week old adult mice were fed a cuprizone or control diet for 5 weeks. At 4 weeks, metformin was given to mice at a dose of 250 mg/kg and 500 mg/kg in their drinking water, and continued for 2.5 weeks. Despite continued cuprizone, some degree of endogenous remyelination begins at approximately 4 weeks, and commences fully upon cuprizone withdrawal. Tissue was collected for analysis 1.5 weeks post-cuprizone withdrawal. **(B)** Representative images of MBP (green) and NF (red) immunohistochemistry in white matter (WM) and gray matter (GM) from control and cuprizone (+/− metformin-treated) mice. Merge is the overlay of MBP and NF images. **(C)** Quantification of mean MBP intensity in white matter. **(D)** Quantification of mean MBP intensity in gray matter. **(E)** Object based colocalization was used to overlay MBP and NF images to get a pseudo readout of myelin sheaths in white matter. **(F)** Object based colocalization was used to overlay MBP and NF images to get a pseudo readout of myelin sheaths in gray matter. Control group (blue bar); cuprizone 4.5 weeks ON (black bars); cuprizone OFF (Cup. OFF) refers to 1.5 weeks following withdrawal of cuprizone chow, with no metformin, i.e., endogenous early remyelination (purple bars); early remyelination with low metformin (250 mg/kg, pink bars), early remyelination with high metformin (500 mg/kg, orange bars). *n = 5* per condition (each mouse represented by closed circles). Statistical analysis was performed using one-way ANOVA with multiple comparisons (DF = 20 within columns). Error bars represent standard error of the mean. *p < 0.05; **p < 0.01; ***p < 0.001.

Adult mice (8 weeks old) were given cuprizone in their chow for 5 weeks to induce demyelination; age-matched control mice were fed a regular diet. At 4 weeks into the cuprizone diet, the timepoint at which OL cell death (and subsequent demyelination) has *already occurred*, and early remyelination is about to commence, mice were administered metformin through their drinking water (with an average daily water intake of 3 mL) at a concentration of either 250 mg/kg (referred to as ‘low metformin’) or 500 mg/kg (referred to as ‘high metformin’; see Figure 8A; Gudi et al., 2014). The concentrations of metformin were determined from studies showing therapeutic efficacy (i.e., reduced blood glucose levels) in diabetic mice (Han et al., 2017). Total metformin treatment duration was for 2.5 weeks to reach a point of “early remyelination” in control mice (1.5 weeks beyond cuprizone withdrawal), a point at which only limited remyelination typically occurs (Gudi et al., 2009).

First, to ensure robust demyelination was occurring, immunohistochemistry using MBP antibodies to detect myelin and neurofilament (NF) antibodies to detect neuronal axons was performed on brain tissue from mice that only received cuprizone infused chow for 4.5 weeks (Cup. ON 4.5 week group). We observed significant decreases in both MBP and NF intensity, in both white matter and gray matter structures, when compared to control mice that were fed a natural diet (Figure 8B, upper two rows, and Figures 8C,D). Upon removing cuprizone to promote endogenous remyelination, we saw that the low and high metformin treatment groups had significantly increased MBP signal intensity when compared to tissue sections taken from mice that underwent endogenous remyelination without metformin (Cup. OFF +2.5 week no metformin group; Figure 8B, lower 3 rows, and Figure 8C). Remarkably, in both white matter (corpus callosum) and gray matter, low and high metformin treated groups showed increased MBP intensities to levels similar to that seen in control mice *without demyelination* (Figures 8C,D). Within the corpus callosum, low metformin treated mice significantly increased their NF signal when compared to those undergoing endogenous remyelination without metformin (Supplementary Figure S4A). However, no significant changes in NF intensities were observed in gray matter (Supplementary Figure S4B).

In addition to individual MBP and NF intensity values, we analyzed the area coverage of MBP and NF signals by thresholding signal intensity equally among the groups. This analysis revealed that, as expected, the MBP+ area was significantly decreased upon cuprizone treatment (Cup. 4.5 week ON group), and was followed by a significant increase in the area of MBP+ regions in both white and gray matter upon removal of cuprizone (Supplementary Figures S4C,D). However, the low and high metformin groups showed an increase in MBP+ area coverage in white and gray matter regions relative to their no metformin, cuprizone-treated controls, with the area of MBP+ white matter being similar to levels seen in the no cuprizone control group (Supplementary Figures S4C,E). The NF+ area on the other hand was only significantly decreased in the white matter of cuprizone 4.5 week mice, with no differences in metformin treatment groups at 2.5 weeks (Supplementary Figures S4D,F). In other words, following cuprizone withdrawal, the no metformin and metformin groups displayed similar levels of NF+ area when compared to no cuprizone controls. However, in gray matter regions, both low and high metformin treatment increased NF+ areas when compared to the no metformin group, with the level of NF+ area in metformin groups being similar to no cuprizone controls (Supplementary Figure S4F).

We also performed object based colocalization to look at the percentage of overlap occurring between axons (NF-positive regions) and myelin (MBP-positive regions). In doing so, we report a pseudo readout for myelin sheaths since the presence of MBP overlapping a NF region would be indicative of myelin wrapping. This analysis revealed that when compared to the demyelinated group (Cup. 4.5 week ON) and the endogenous remyelination group (Cup. OFF + no metformin), both low and high metformin groups significantly increased the percentage of MBP and NF overlap in the white matter (Figure 8E). In the gray matter regions however, no significant differences were found (Figure 8F).

### Metformin promotes oligodendrocyte differentiation during recovery from cuprizone-mediated demyelination

**Figure 9:**
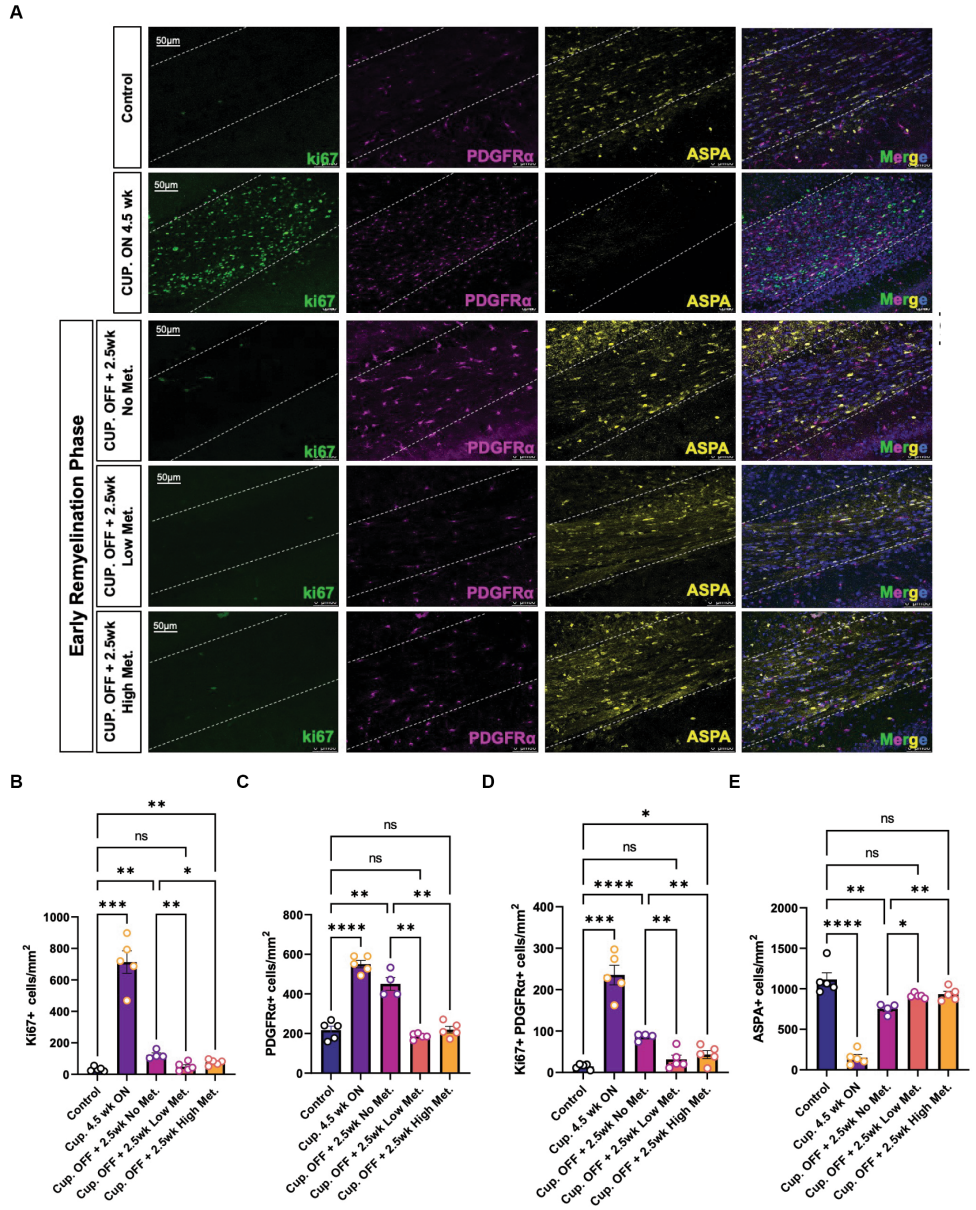
Metformin alters oligodendrocyte cellular dynamics during remyelination. **(A)** Representative image of control, demyelinated, and early remyelinated corpus callosum. PDGFRα antibodies (magenta) were used to label OPCs and ASPA antibodies (yellow) were used to label mature OLs. ki67 (green) was used to detect proliferative cells. **(B)** Quantification of Ki67+ cells per area **(C)** Quantification of PDGFRα+ cells per area. **(D)** Quantification of double positive Ki67+ PDGFRα+ cells per area. **(E)** Quantification of ASPA+ cells per area. Control group (blue bara); cuprizone 4.5 weeks ON (purple bars); cuprizone OFF, 2.5w no metformin (magenta bars); cupriozone OFF, 2.5w low metformin (250 mg/kg, orange bars); cuprizone OFF, 2.5w high metformin (500 mg/kg, yellow bars). Statistical analysis was performed using one-way ANOVA with multiple comparisons (DF = 20 within columns).*n = 5* per condition. Error bars represent standard error of the mean. *p < 0.05; **p < 0.01; ***p < 0.001.

The enhanced recovery observed under metformin treatment (Figure 8) could be due to changes in OPC and OL cellular dynamics such as proliferation and/or differentiation. Using immunohistochemistry, we detected OPCs with PDGFRα antibodies, mature OLs with ASPA antibodies, and proliferating cells with ki67 antibodies (Figure 9). We found that treatment with cuprizone for 4.5 weeks significantly increased the presence of both ki67+ (Figure 9B) and PDGFRα+ cells (Figure 9C), and decreased the amount of ASPA+ cells (Figure 9E), relative to that seen in age matched controls. As expected, when the cuprizone diet is removed to allow for endogenous remyelination, the number of ki67+ and PDGFRα+ cells significantly decreased, while the number of ASPA+ cells increased (Figures 9B,C,E). Additionally, the number of proliferating PDGFRα cells (double positive for ki67 and PDGFRα) drastically increased in the cuprizone only group, and decreased significantly after removal of cuprizone (Figure 9D). Remarkably, when mice were treated with low and high metformin in their drinking water, a significant increase (compared to the cuprizone only group) in ASPA+ OLs and decrease in PDGFRα+ OPCs occurred, which similar to our *in vitro* findings, suggested that OPCs accelerate their differentiation timeline with metformin treatment. This finding is in agreement with the increased MBP intensities and MBP/NF colocalization seen in early remyelinating white matter in response to metformin treatment (Figure 8).

The greatest decrease in proliferating cells were seen in the low and high metformin groups when compared to the cuprizone off + no metformin group (Figures 9B,D). However, metformin’s potential impact on cell death was also important to consider when interpreting the effects of metformin on OL dynamics. To assess cell death, we used the terminal deoxynucleotidyl transferase dUTP nick end labeling (TUNEL) method to fluorescently label the 3′-hydroxy terminal of the double strand DNA breaks generated during apoptosis (Darzynkiewicz and Zhao, 2011). We found that the only changes in TUNEL+ cells were observed in the cuprizone only group (Supplementary Figure S5), suggesting that metformin does not alter OPC or OL survival, at least during the treatment period, which begins at week 4, after the period in which cuprizone-mediated death is known to occur (Gudi et al., 2009).

### Astrocytes and microglia remain grossly unchanged with metformin treatment during recovery from cuprizone-mediated demyelination

**Figure 10:**
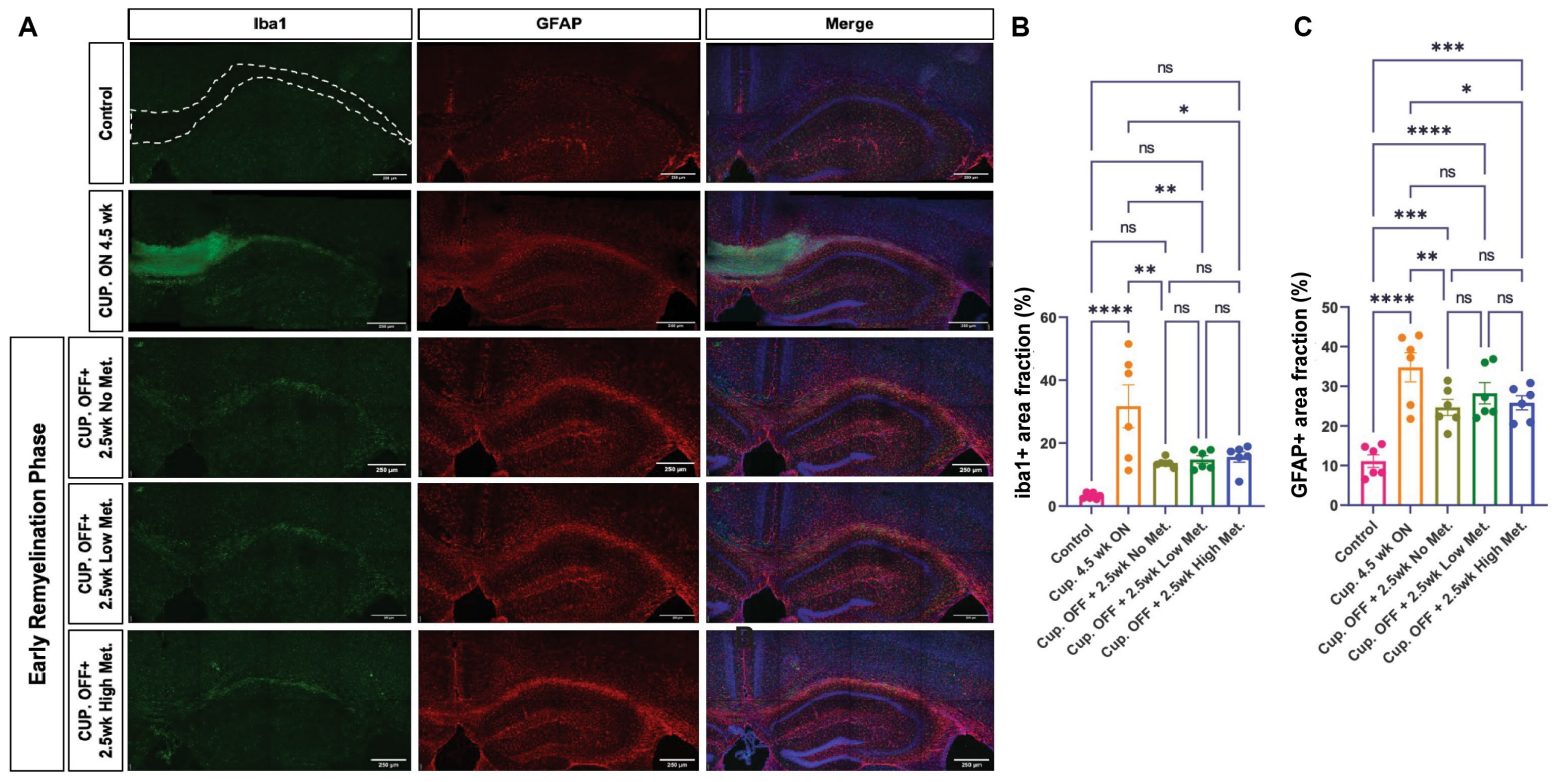
Metformin does not alter Iba1+ and GFAP+ cell areas during remyelination. **(A)** Representative images of Iba1+ cells (green) and GFAP+ cells (red), including DAPI nuclear stain (blue) in the corpus callosum (dotted line) and adjacent areas. *Scale bar*, 200 microns. *n = 6* per condition. Area coverage of Iba1+ cells **(B)** and GFAP+ cells **(C)** in the corpus callosum. Control group (pink bars); cuprizone 4.5 weeks ON (yellow bars); cuprizone OFF, 2.5w no metformin (olive bars); cuprizone OFF, 2.5w low metformin (250 mg/kg, green bars); cuprizone OFF, 2.5w high metformin (500 mg/kg, blue bars). Statistical analysis was performed using one-way ANOVA with multiple comparisons (DF = 25 within columns). Scale bar, 200 microns. *n = 5* per condition. Error bars represent standard error of the mean.

Myelin repair depends on oligodendroglia, but astrocytes and microglia also play a role in the remyelination process by contributing to myelin clearance and by modulating the injury-driven inflammatory response (Traiffort et al., 2020). Metformin has been shown to have immune modulatory effects and therefore could be affecting astrocyte and microglia dynamics. We therefore performed immunohistochemistry to detect microglia and astrocytes using the pan-microglia marker Iba1 and the GFAP marker, respectively (Figure 10). A stark elevation in Iba1+ and GFAP+ areas were noted within the corpus callosum of the cuprizone-treated group (Figures 10B,C). Upon cessation of the cuprizone diet, however, Iba1 coverage was significantly decreased in the no, low, and high metformin groups when compared to the cuprizone control group, but microglia in the remyelination groups did not return to the baseline Iba1 levels seen in the controls (Figure 10B). Similarly, GFAP+ area was decreased during the early remyelination phase in the no, low, and high metformin groups when compared to cuprizone control mice (Figure 10C). The levels of GFAP did not return to the baseline amounts saw in the control group. Overall, metformin did not affect either Iba1+ or GFAP+ areas during the remyelination phase.

## Discussion

To investigate the functional relationship between oligodendroglial metabolic state and myelination, we used the AMPK activator metformin to assess how changes in OL energetics coincide with key stages in OL lineage progression, both during normal development and during repair following myelin damage in young adult mice. We found that healthy OLs treated with metformin at the physiologically relevant concentration of 200 μM, or with the supra pharmacological concentrations of 500 μM and 1 mM *in vitro*, exhibited a linear, dose dependent increase in AMPKα activation that resulted in changes in OXPHOS and glycolysis at multiple stages during oligodendroglial lineage progression. In cultures differentiated for 1 day, which are still predominantly OPCs, OXPHOS was *reduced* by higher concentrations (500 μM and 1 mM) of metformin, while glycolysis was significantly *increased*. Tellingly, metformin also induced the largest fold increase in OL differentiation at this early time point, suggesting that OPCs are particularly responsive to metformin’s influence on differentiation. Interestingly, treatment with 1 mM metformin induced a glycolytic switch in OLs by enhancing glycolysis and downregulating OXPHOS (from day 1 through day 7). Remarkably, this glycolytic switch can still promote OL differentiation, albeit not at the same degree as OLs treated with 200 μM and 500 μM metformin, where a noticeable *increase* in both OXPHOS and glycolysis confers an enhanced bioenergetic phenotype (and is most effective at promoting OL differentiation). One possibility is that this pro-differentiation effect could be due to changes in the pentose phosphate pathway (PPP), which is in part reliant on the level of glycolysis occurring in the cell and is necessary for OL health as inhibition of PPP in OLs with supra concentration of the G-6-P inhibitor dehydroepiandrosterone (DHEA) compromises NADPH supply to cause significant OL death and decreases in myelin (Kilanczyk et al., 2016).

Using a knockdown approach to prevent expression of AMPK catalytic subunits, we reported that metformin requires an active AMPK complex to promote differentiation, confirming a previous report (Neumann et al., 2019). To additionally interrogate whether metformin’s ability to promote differentiation also required metformin’s ability to stimulate cellular bioenergetics, we inhibited both glycolysis and OXPHOS using 2-DG and oligomycin, respectively. Under these conditions metformin treatment did *not* promote OL differentiation, suggesting that metformin requires active glycolysis and/or OXPHOS to potentiate OL differentiation. It should be noted that only aged adult OPCs, not young adult OPCs, were found to differentiate more effectively in response to metformin in the above-mentioned study by Neumann et al. (2019). In contrast, our study found that juvenile OPCs (approximately 14 days in vitro from cultures prepared at postnatal day 1 or 2) respond to metformin to accelerate their differentiation, also in an AMPK dependent fashion, suggesting that metformin’s effects on OPCs may be highly age- or timing-dependent. One difference may be that in the Neumann study, young adult OPCs were treated for 5 days with metformin prior to analysis for differentiation, while in the current study juvenile OPCs were analyzed at day 1, day 3, and day 5, with the maximal response to metformin observed at 24 h. One interpretation is that juvenile OPCs exhibit a greater ability to respond to metformin relative to that in young adults, however it remains to be determined if young adult OPC may be able to increase their differentiation in response to metformin after 24 h, as was observed in juvenile OPCs.

Our *in vivo* investigation showed that metformin treatment in mice after cuprizone-induced demyelination, significantly enhanced OL differentiation to promote remyelination, with densities of OLs approaching that of controls (that seen in mice that did not undergo cuprizone-mediated demyelination). Furthermore, no differences in cell death were observed nor were microglia or astrocyte populations grossly changed in the metformin-treated groups compared to mice undergoing remyelination without metformin. This could be because the large increase in microglia and astrocyte that is observed during cuprizone-mediated demyelination takes place much earlier, i.e., during the first few weeks of cuprizone treatment during the peak of OL death and myelin debris clearance (Gudi et al., 2014). However, the results from cell culture experiments indicate that metformin is able to directly influence OPC/OL dynamics, at least in the context of our treatment paradigm in which we deliver metformin largely during myelin repair.

One puzzling aspect of metformin’s effect was the different response seen in white matter versus gray matter. In white matter, but not gray matter, the MBP-NF colocalization was significantly improved by metformin, so much so that the degree of colocalization was no longer different from that seen in mice without cuprizone demyelination (Figure 9). However gray matter regions in MS are reported to experience more robust remyelination compared to white matter, however this is proported to be due to the absence of infiltrating leukocytes (such as T-cells and neutrophils), which would not be factors in the cuprizone model (Prins et al., 2015). Another possibility is that microglia could present with a different inflammatory profile in demyelinated gray matter lesions, which upon metformin treatment could be modulated given that several studies have linked metformin to decreases in pro-inflammatory microglia (Abdi et al., 2021). It is possible that white matter and gray matter repair may not follow the same “rules” following cuprizone-mediated demyelination, which occurs largely devoid of leukocyte infiltration, but it will be interesting to investigate in other models whether metformin treatment shows a preference for enhancing white matter rather than gray matter remyelination.

Additional work is also needed to establish the extent and limitations of metformin’s ability to influence OL dynamics more broadly in disease. However, there are already some clues as to the potential benefit to diseases beyond MS. In a rat model of type II diabetes, white matter lesions (often characterized by the loss of OLs) are present before the onset of diabetes-associated-cognitive-decline and, interestingly, when these rats undergo treatment with metformin, white matter lesions are reduced and diabetes-associated-cognitive-decline improved although not to the level of wild type controls (Li et al., 2019). Additionally, a prospective cohort study studying the effect of dementia (a condition associated with white matter loss) in the type II diabetes population found that patients taking metformin to manage their diabetes *decreased* their risk of dementia by 24% when compared to type II diabetic individuals that were not on any anti-diabetic medications (Hsu et al., 2011). One possibility is that metformin-induced improvements in cognitive function could in part be mediated through changes in OL dynamics in the CNS.

AMPK function in the context of myelination is likely to be highly complex, as many of the known actions of AMPK do not easily fit with a “pro-myelination” phenotype. For instance, active AMPK in the nucleus of hepatocytes is known to *inhibit* the transcription of gluconeogenic and lipogenic genes, and if a similar response were to occur in OLs, that would be predicted to inhibit myelination. Additionally, AMPK activity can regulate cellular growth and proliferation through *suppression* of the mechanistic target of rapamycin (mTOR) pathway (Garcia and Shaw, 2017), and mTOR signaling is well established as *promoting* OL differentiation and remyelination (Tyler et al., 2009; Mather et al., 2022). mTOR consists of two complexes, mTORC1 and mTORC2, both of which are capable of integrating nutrients such as glucose, oxygen, amino acids, and insulin to influence growth and proliferation (Sabatini, 2017). mTORC1 is functionally dependent on the regulatory associated protein of mTOR (raptor) subunit whereas, mTORC2 activity is modulated by the rapamycin insensitive companion of mTOR (rictor) subunit, and both have distinct contributions to CNS oligodendrocyte development, myelination, and myelin repair (Bercury et al., 2014; Lebrun-Julien et al., 2014; Wahl et al., 2014; Musah et al., 2020; Ornelas et al., 2020; Jeffries et al., 2021; Khandker et al., 2022; Dahl et al., 2023). However it should be noted that preventing expression of a *suppressor* of mTOR signaling, Tuberous Sclerosis Complex 1 (TSC1), surprisingly led to hypomyelination during development (Carson et al., 2012; Lebrun-Julien et al., 2014; Jiang et al., 2016) but an enhancement of myelin repair in adult mice (McLane et al., 2017), suggesting that loss of appropriate mTOR suppression may be either anti- or pro-myelination, depending on the context. Future studies are needed to fully understand the interactions between AMPK and mTOR in the context of metformin administration.

In the current study we report that metformin enhances AMPK activity to increase glycolysis in OLs, that metformin-induced changes in AMPK activation are necessary for metformin to promote OL differentiation, and that metformin-induced differentiation is dependent on functional metabolic pathways. Early stages of myelin repair were strongly affected by metformin, with OPC densities and OL densities being negatively and positively regulated, respectively. While enhanced clearance of myelin cannot be ruled out, as metformin has previously been demonstrated to promote M2-like phenotype in macrophages to enhance myelin clearance following peripheral nerve injury (Zhou et al., 2023), we did not observe the altered microglial cell area coverage that would be characteristic in a change in activation status, and, most important, we administered metformin well after the peak of debris clearance. Together our findings contribute to the growing body of knowledge that indicates that metformin, in addition to its known role in modulating neuroinflammation, may independently modulate oligodendroglial dynamics to promote successful myelin repair.

## Data availability statement

The original contributions presented in the study are included in the article/Supplementary material, further inquiries can be directed to the corresponding author.

## Ethics statement

The animal study was approved by Stony Brook University Institutional Animal Care and Use Committee. The study was conducted in accordance with the local legislation and institutional requirements.

## Author contributions

HC and MN contributed to conception and design of the study. MN, MA, and HC wrote the manuscript. MN, MA, MU, and AV performed experiments. MN, MA, AV, and HC analyzed data. All authors contributed to the article and approved the submitted version.
