# Supplementary material for: The AMPK activator metformin improves recovery from demyelination by shifting oligodendrocyte bioenergetics and accelerating OPC differentiation
Source: Front Cell Neurosci. 2023 Oct 12;17:1254303. doi: 10.3389/fncel.2023.1254303 (PMC10613472; doi:10.3389/fncel.2023.1254303)
Supplement: Supplementary file 1 [file Data_Sheet_1.PDF]

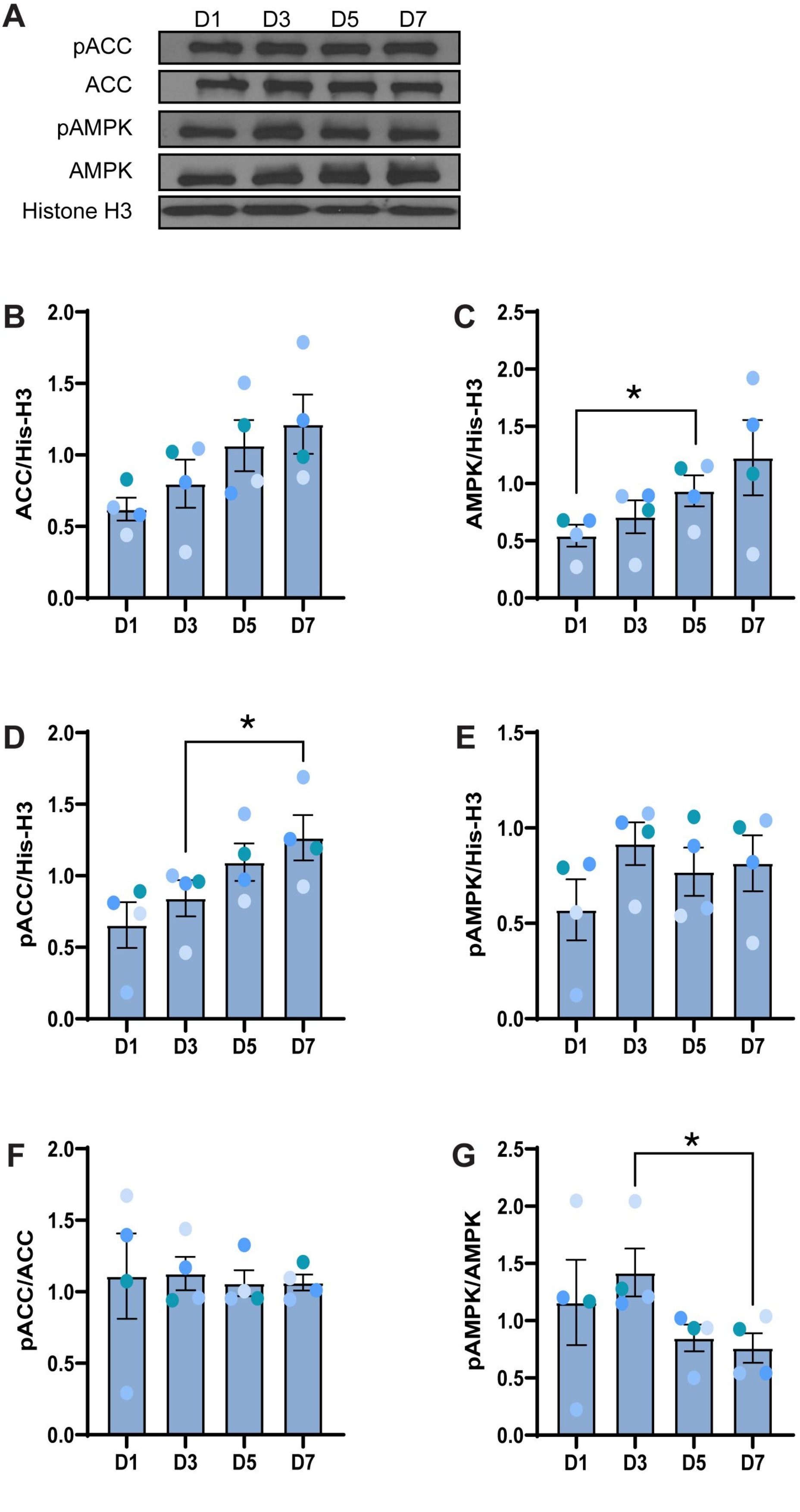

Supplementary Figure 1

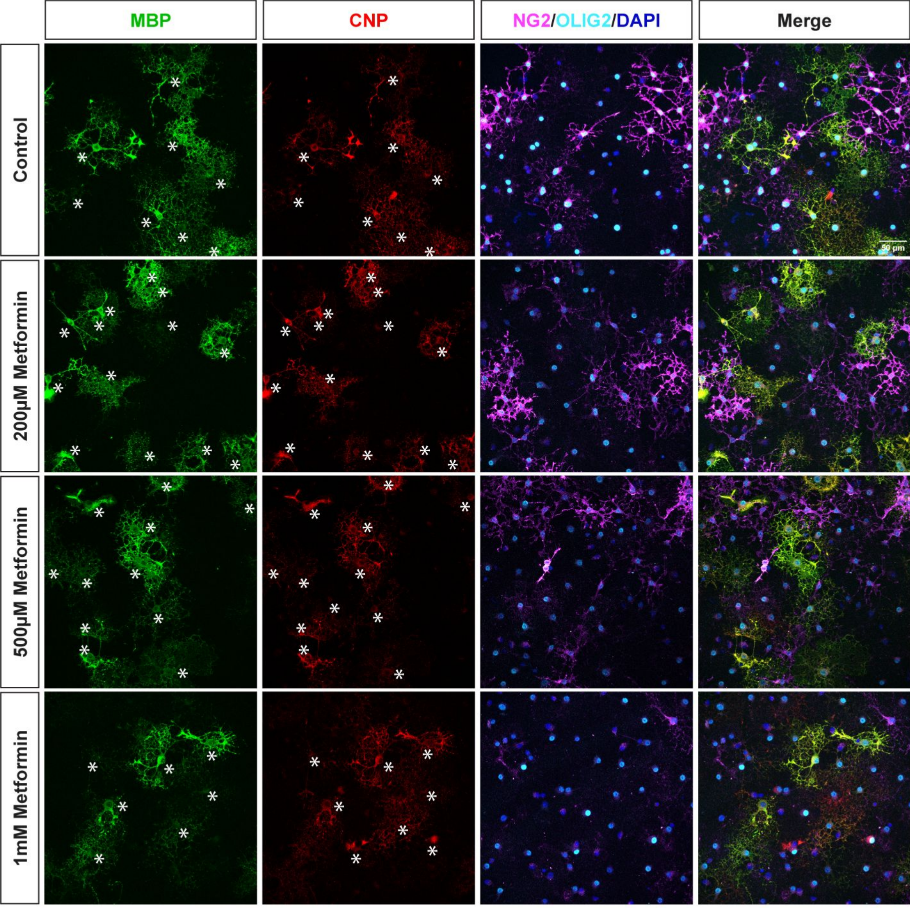

Supplementary Figure 2

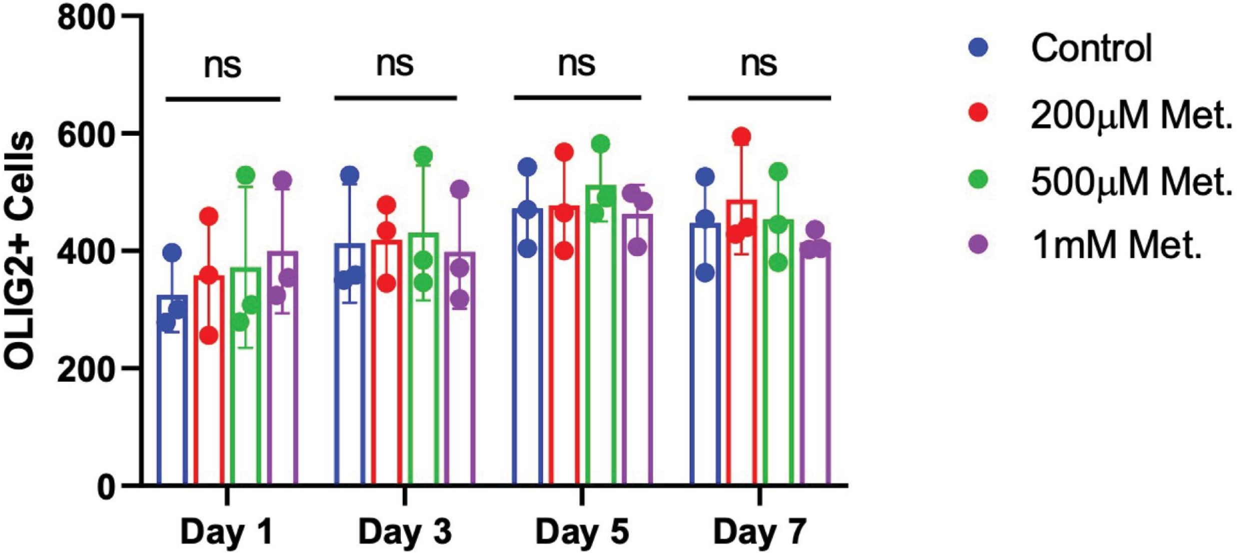

Supplementary Figure 3

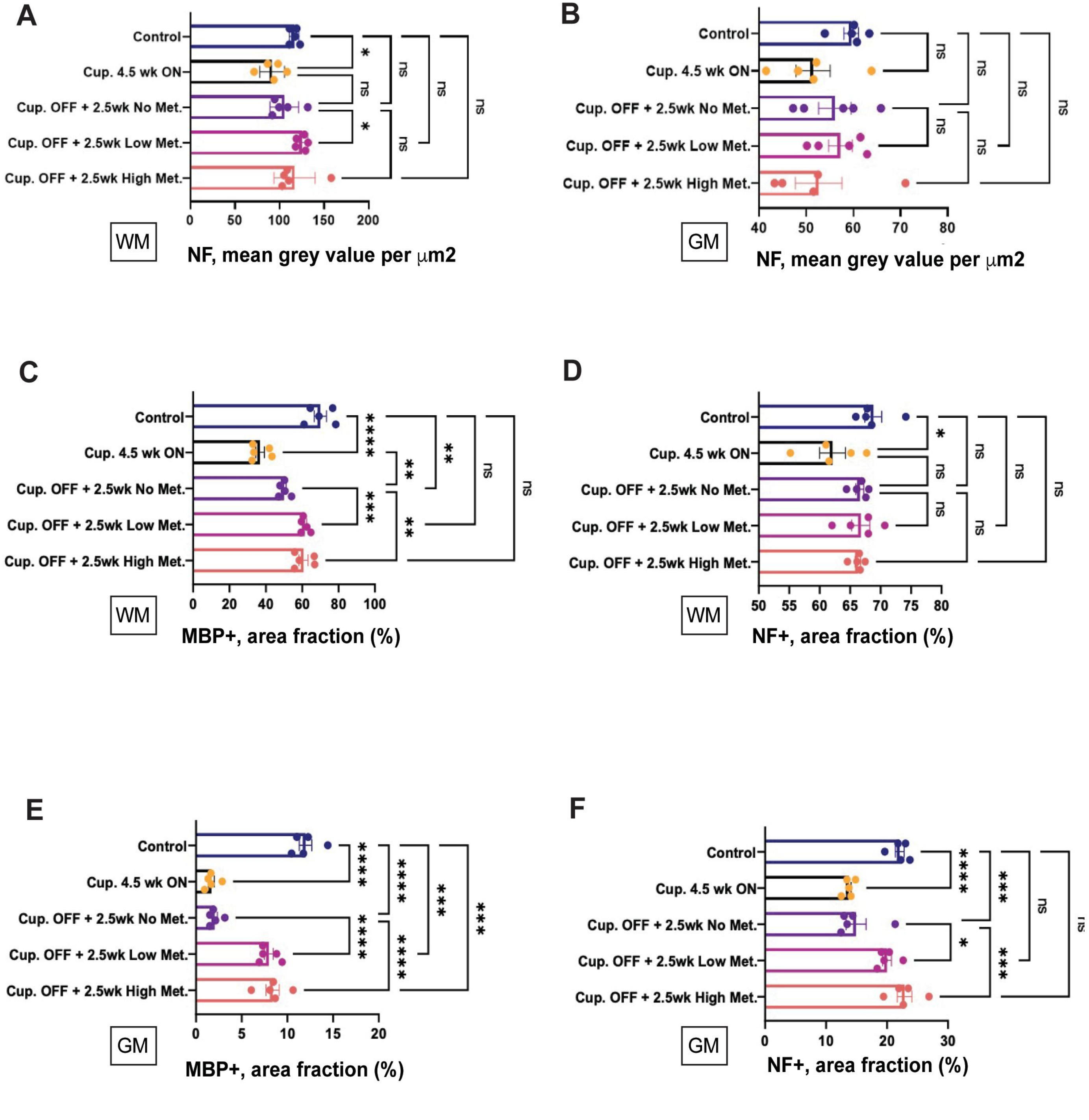

Supplementary Figure 3

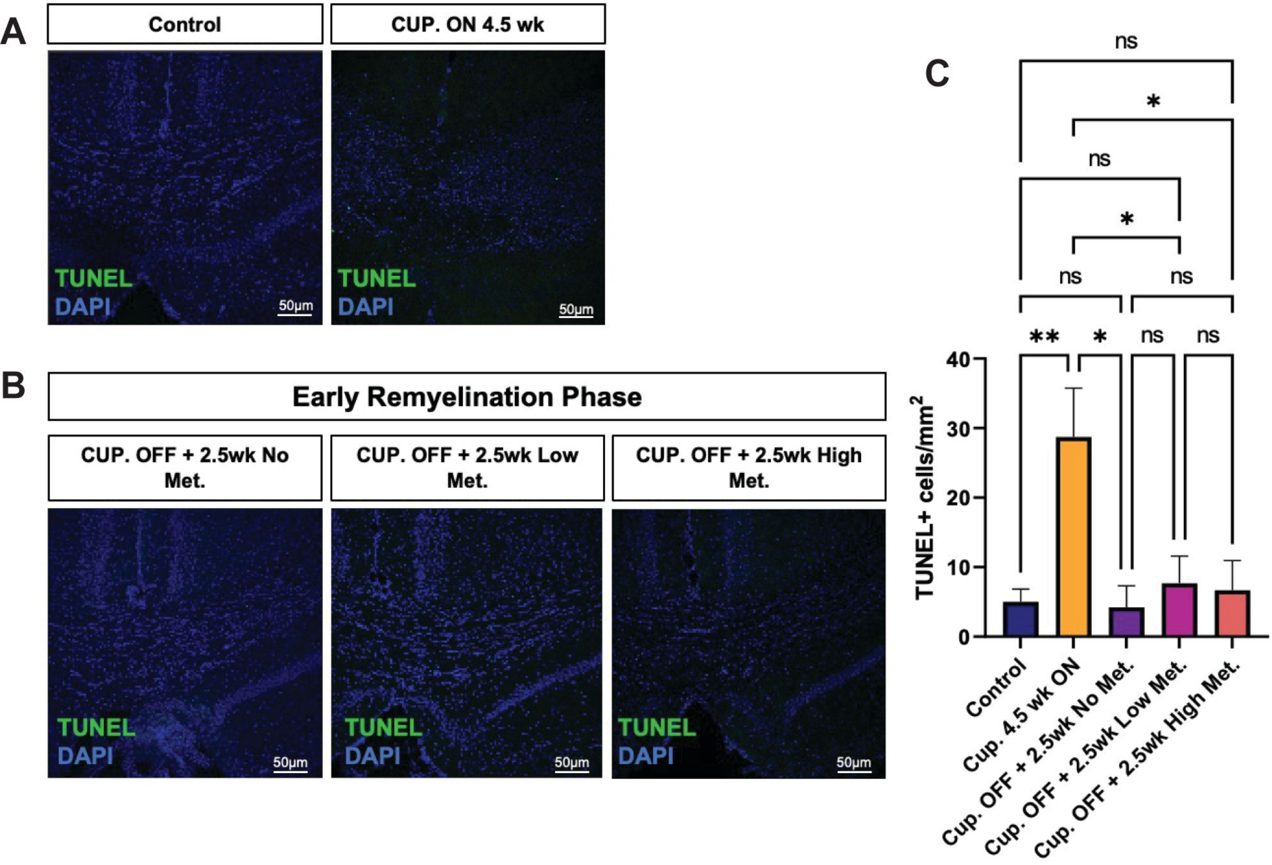

Supplementary Figure 4

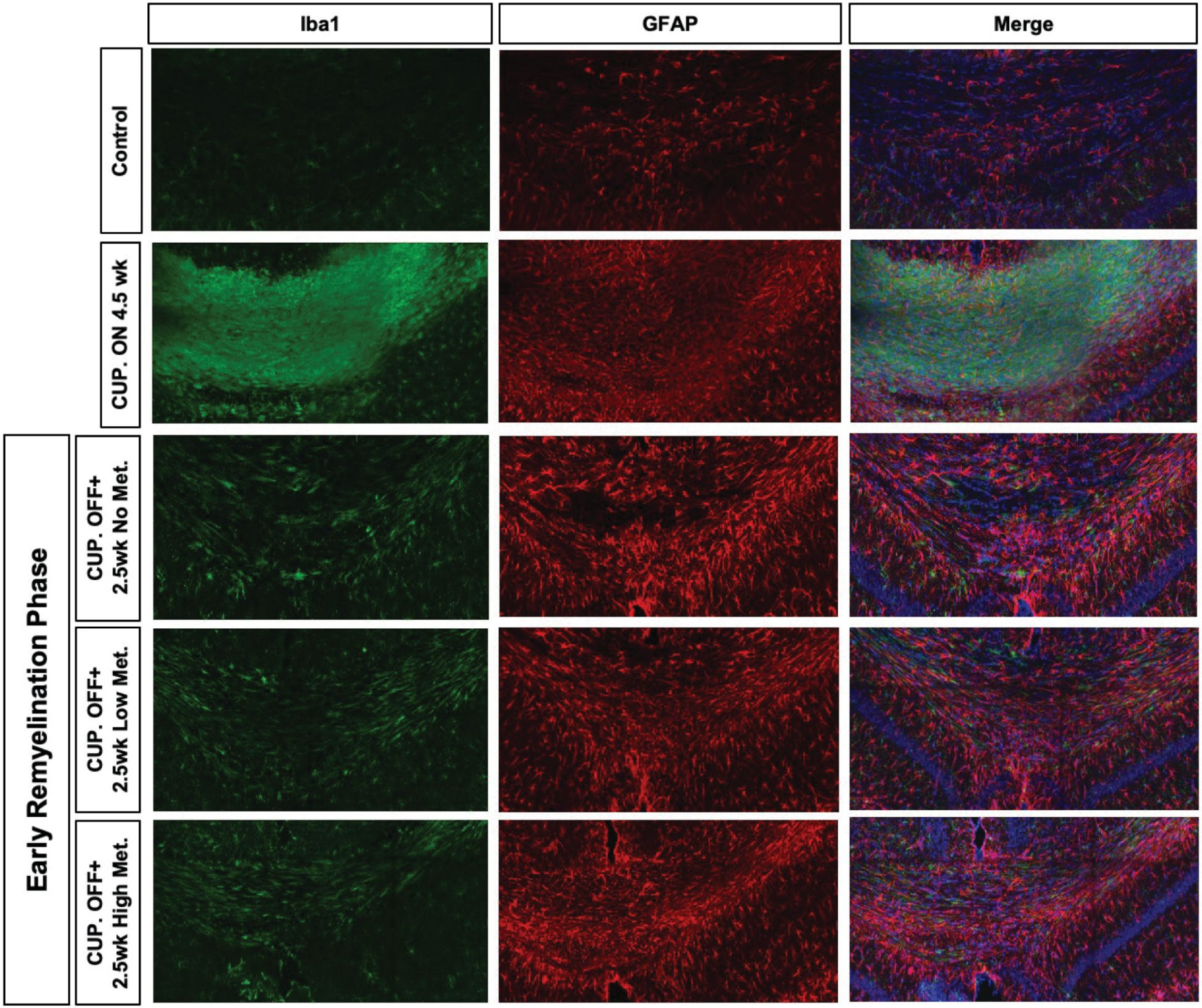

Supplementary Figure S6
